# A robust E learning recommendation system based on novel interval valued bipolar fuzzy hypersoft set theory

**DOI:** 10.1038/s41598-026-42231-6

**Published:** 2026-03-12

**Authors:** Muhammad Imran Harl, Muhammad Saeed, Muhammad Haris Saeed, Muhammad Salman Habib, Mehran Ullah

**Affiliations:** 1 Department of Mathematics, University of Management and Technology, Lahore, 54700 Punjab Pakistan; 2 Department of Chemistry, University of Management and Technology, Lahore, 54700 Punjab Pakistan; 3 Institute of Knowledge Services, Center for Creative Convergence Education, Hanyang University ERICA Campus, Ansan, Gyeonggi-do 15588 South Korea; 4 School of Business and Creative Industries, University of the West of Scotland, Paisley, PA1 2BE UK

**Keywords:** Soft set theory, Fuzzy set theory, Optimization, Bipolar soft Set, Bipolar hypersoft set, Decision support systems, Decision making, Applied mathematics, Computational science, Pure mathematics

## Abstract

Understanding bipolar information is crucial as it enables individuals to make informed decisions that consider both extremes of a spectrum, leading to more balanced and effective outcomes. Interval-valued bipolar fuzzy set (IVBFS) has already been introduced in the literature as a great decision-making tool that can capture interval-valued bipolar information to properly address uncertainty. In this article, we introduce a hybrid of Interval-valued bipolar fuzzy set (IVBFS) and bipolar hypersoft sets (BHSS) called interval-valued bipolar fuzzy hypersoft set $(IVBF_{HSS})$, which merges the capabilities of IVBFS and BHSS. The rationale behind the design of the presented data structure is to manipulate and process information in decision-making scenarios when the data is bipolar, has multiple attributes that need to be addressed up to a sub-attributive level to get a proper representation of the data provided, and needs to be presented in the form of intervals. In $(IVBF_{HSS})$, two hyper soft sets (HSSs) are used, one providing positive interval-valued membership information and the other providing negative interval-valued membership information. We outline the essential features and basic operations of $(IVBF_{HSS})$ in this paper, examining its commutative, associative, distributive, and De Morgan laws to ensure a comprehensive analysis. To demonstrate the significance of $(IVBF_{HSS})$, we develop a preferential decision support algorithm for selecting the best alternative in e-learning, such as identifying the most suitable instructional method, which can effectively be formulated as a Multi-Attribute Decision-Making (MADM) problem. This approach allows for the systematic evaluation of various alternatives based on multiple parameters and sub-parameters, enabling a rational and well-informed decision. This algorithm helps select the best alternative from a given set of options, leveraging the versatile nature of $(IVBF_{HSS})$. The presented study conducts both computation-based and structural comparisons to evaluate the adaptability and reliability of the proposed framework.

## Introduction

Numerous mathematical strategies have been suggested by scholars to cope with uncertainties. Fuzzy sets (FS) and membership degrees of alternatives were first proposed by Zadeh^1^ in 1965. Intuitionistic fuzzy set (IFS), which is a direct extension of FS, was proposed by Atanassov^2^ in 1986. The soft set theory (SS), introduced by Molodtsov^3^ in 1999, provides a mathematical framework for handling uncertainties and vagueness in decision-making and modeling. Unlike traditional uncertainty theories such as probability theory (PT)^4^, FS theory, IFS theory, and rough set theory (RS)^5^, SS theory does not aim to generalize or extend these theories. Instead, it introduces a new concept and approach to deal with uncertainties. The inception of SSs sparked significant interest among researchers, who recognized its potential and began exploring its fundamental properties, hybrid structures, and interactions with other disciplines. The basic properties of SS theory, laying the foundation for further investigations, were presented by Maji^6^ in 2003. However, an important point, arguing that some features of SSs are discussed by Maji, was raised by Ali^7^ in 2009. Ali introduced novel formal properties of SSs and examined the applicability of De Morgan’s laws to SSs. Ali’s work sheds light on the specific characteristics and properties of soft sets that might deviate from conventional set theory. By identifying the limitations and exploring alternative properties, he contributed to a deeper understanding of the SS theory and its distinctive nature. The concept of fuzzy soft sets (FSSs) was introduced by Roy^8^ in 2007, which extends the capabilities of soft sets by incorporating fuzziness. FSSs provide a framework to model and solve decision-making cases that involve both uncertainty and vagueness, allowing for more flexible and nuanced representations of information. FSSs have been applied in game theory^9^ to model decision-making processes, especially in scenarios where players have imprecise preferences or incomplete information. In forecasting^10^, FSSs are used to model uncertain data and make predictions about future trends or events, enabling decision-makers to anticipate potential outcomes more accurately. The deployment of quality functions^11^ involves FSSs to translate customer requirements into product design specifications. FSSs^12^ aid in supplier selection problems by considering multiple criteria with uncertain or incomplete information, facilitating the identification of optimal suppliers based on various factors. FSSs are used in image processing to produce selectively colored images^13^, where different parts of an image are colored based on certain criteria or preferences. In counter-terrorism efforts, FSSs are used to analyze complex networks of terrorist organizations^14^, identify key nodes, and disrupt their activities. FSSs^15^ provide a framework for quantifying and managing uncertainty in decision-making processes, allowing decision-makers to assess the reliability of their conclusions and make informed decisions. FSSs are used in medical diagnosis^16^ to handle incomplete or imprecise patient data, helping healthcare professionals make accurate diagnoses and treatment decisions.

Similar to these approaches, soft set hybrids with neutrosophic and rough sets have been reported in literature to address uncertainty as well. Bui et al. applied a hybrid of sequenced soft and neutrosophic sets for medical diagnostic applications^17^. Similarly, Bui et al. also devised an intuitionistic fuzzy rough soft set approach for handling uncertainty, vagueness, and ambiguity in complex decision-making environments with particular focus on a series of applications including agricultural land evaluation and educational support^18^. Furthermore, an alternative approach to solving decision-making problems based on FSS was proposed by Alcantud^19^ in 2017. His approach likely offers different perspectives or methodologies for utilizing FSSs in decision-making processes. After that, the concept of interval-valued fuzzy soft sets(IVFSSs) was introduced by Yang^20^ in 2009. IVFSSs provide a framework for handling uncertainties and vagueness in decision-making scenarios, where both the membership degrees and the characteristic function values are represented as intervals. This allows us to have a more flexible and robust representation of imprecise and uncertain information. In decision-making, considering bipolar information allows for a more comprehensive analysis and evaluation of the problem at hand. This duality is commonly observed in various fields, including medical, social, and business domains. In the medical field, there may be decisions to be made regarding treatment options, where one choice involves artificial interventions (such as medication or surgery) while the other emphasizes natural or alternative therapies. In business and finance, decision-making often involves assessing the potential gains and losses associated with different investment opportunities. Considerations may include evaluating potential profit margins, financial risks, market conditions, and trade-offs between short-term gains and long-term sustainability. Based on these facts, a generalization of the idea of FS, bipolar-valued fuzzy set (BVFS) was first proposed by Zhang^21^ in 1994, and the basic operation of BVFS was presented by Lee^22^ in 2000. The idea of a bipolar soft set (BSS), which is a hybrid of bipolarity^23^, and SS was independently developed by Shabir^24^ in 2013.

Fuzzy bipolar soft sets (FBSS) were introduced by Naz^25^ in 2014, and additionally, he addressed their algebraic fundamentals and applications. Bipolar fuzzy soft sets (BFSS) were first introduced by Abdullah^26^ in 2014, also investigating their use in decision-making problems. The idea of an interval-valued bipolar fuzzy weighted neutrosophic set (IVBFWNS) was developed by Deli^27^ in 2016, by applying the IVBFS to neutrosophic sets (NSs). Hamacher aggregation operators based on IVBFS were developed by Wei^28^ in 2019, further examining their salient characteristics. Interval-valued bipolar fuzzy ideals (IVBFIs) were proposed by Pairote^29^ in 2021. Its objective was to implement the IVBFS theory to address the algebraic structure of semigroups. The notion of bipolar fuzzy soft topology (BFS-topology) and bipolar fuzzy soft mappings, as proposed by Riaz^30,31^, introduces a mathematical framework that combines bipolarity, fuzzy sets, and soft sets to address topology-related concepts and decision-making problems. Hypersoft set (HSS) introduced by Samarandache^32^ in 2018, transforming the function F into a multi-attribute function. As a generalization of SS, HS is more flexible than SS and is better suited for challenges involving decision-making. The basic and essential properties of HSS were introduced by Saeed^33–35^ in 2020 and 2021.

Fuzzy Hypersoft Sets (FHSS) was proposed by Yolcu^36^ in 2021, which provided correct options in challenges involving decision making. Intuitionistic fuzzy hypersoft set (IFHSS) with basic operation was defined by Yolcu^37^ in 2021. Picture fuzzy hypersoft set (PFHSS) initiated by Saeed^38^ in 2023, which is a hybrid structure of HSS and picture fuzzy set (PFS)^39^. A decision-making technique specifically designed for the IFHS environment was proposed by Zulqarnain^40^ in 2020. The interval-valued complex fuzzy hypersoft set IVCFHS was first introduced by Rahman^41^ in 2021, with some basic operations. The correlation-based TOPSIS strategy for $P_{y}FHSS$ developed by Zulqarnain^42^ in 2021 used their proven method to choose the best face mask. The development of aggregation operators (AOs) specifically designed for $P_{y}FHSS$ was developed by Siddique in 2021^43^. These AOs were designed to facilitate the combination of information and data within the $P_{y}FHSS$ framework. The concept of q-rung orthopair fuzzy hypersoft sets, introduced by Khan^44^ in 2022, extends the traditional orthopair fuzzy hypersoft set framework by incorporating the notion of q-rung orthopair fuzzy sets. This extension improves the modeling and analysis of decision-making problems by considering various interactions between input arguments.

By incorporating the rough approximation of the FHSS into the supplier selection process introduced by Rahman^45^ in 2022, the aim was to improve the accuracy and robustness of the decision-making outcomes. Arshad^46^ framework in 2024 for selecting optimal COVID-19 masks based on aggregations of interval-valued multi-fuzzy hypersoft sets (IVMFHSS) offers a robust and innovative approach to decision-making in the context of pandemic response. Picture fuzzy hypersoft graph (PFHSG) developed by Saeed^47^ in 2023 provides new information on product sale risk analysis with a pictorial representation of its associated factors. Bipolar hypersoft set (BHSS) introduced by Musa^48^ in 2021, which is a direct extension of HS. BHSS has applications in various domains, such as decision analysis, multi-criteria decision making, risk assessment, and opinion mining, among others.

By considering the bipolar nature of the elements and incorporating fuzzy or uncertain information, bipolar hypersoft sets offer a powerful tool to handle decision problems with conflicting or contrasting aspects. Topological Structures via BHSS was introduced by Musa^49^ in 2022, which was defined on collections of BHSS. The concept of q-rung orthopair fuzzy hypersoft sets, as introduced by Khan^44^ in 2022, extends the traditional orthopair fuzzy hypersoft set framework by incorporating the notion of q-rung orthopair fuzzy sets. This extension improves the modeling and analysis of decision-making problems by considering various interactions between input arguments.

The concept introduced by Surya^50^ in 2024, namely the notion of q-rung linear diophantine fuzzy hypersoft set, which is capable of handling multi-sub-attributed q-rung linear diophantine fuzzy situations in the real world. Zain^51^ in 2024 focused on developing a dam suitability map and identifying potential dam sites. This was achieved using a hybrid model that combines a fuzzy hypersoft set with a plithogenic multi-polar fuzzy hypersoft set.

### Research gap

Although e-learning platforms have grown rapidly, current recommendation systems still struggle to capture the complex preferences of learners. Traditional approaches, such as collaborative filtering or basic fuzzy set methods, focus primarily on positive feedback and often overlook negative preferences, which can lead to recommendations that do not fully match the actual interests of learners. The learners’ choices are often uncertain or hesitant, particularly when dealing with different types of content or learning strategies, yet existing systems cannot represent such preferences based on intervals or partially known information. Furthermore, e-learning materials are multidimensional, with sub-attributes like difficulty, engagement, interactivity, and format, but conventional systems usually consider only broad attributes, missing finer details of learner needs. The lack of advanced frameworks, such as Interval Valued Bipolar Hypersoft Sets ($IVBF_{HSS}$), prevents simultaneous modeling of positive and negative preferences while addressing uncertainty and hierarchical attribute relationships. Therefore, there is a need for improved recommendation systems that can handle these complexities and provide more accurate, personalized learning suggestions (Table 1).

**Table 1:** Comparative evaluation of fuzzy, soft, hypersoft, and $IVBF_{HSS}$ approaches in E-learning recommendation systems.

Feature	Fuzzy sets	Soft sets	Hypersoft sets	$IVBF_{HSS}$ (Proposed)	Advantage for E-learning
Preference Type	Only positive membership	Only positive membership	Can handle multi-attribute data	Handles both positive and negative preferences	Can model likes and dislikes simultaneously, capturing true learner sentiment
Uncertainty	Single value, limited uncertainty	Single or simple set-based uncertainty	Multi-attribute uncertainty possible	Interval-valued representation	Can represent hesitation and partial knowledge in learner preferences
Attribute Structure	Flat, single-level	Simple attributes	Multi-level attributes	Multi-level attributes with sub-attributes	Captures hierarchical and detailed aspects of learning content
Multi-Dimensional Content	Limited	Limited	Supports multi dimensional content	Fully supports multi dimensional, hierarchical content	Models complex e-learning content more accurately
Decision Making	Simple aggregation	Basic decision rules	Supports complex aggregation	Supports advanced bipolar and interval-based aggregation	Produces robust, personalized recommendations considering both positive and negative feedback
Suitability for E-Learning Recommendations	Basic recommendations	Limited personalization	Improved personalization	High personalization and context-awareness	Best suited for handling nuanced learner preferences and multi-faceted learning materials

### Main objectives

This study aims to overcome the limitations of existing e-learning recommendation systems by exploring the use of $IVBF_{HSS}$. It examines how $IVBF_{HSS}$ can improve the accuracy and personalization of recommendations by representing both positive and negative learner preferences, handling uncertainty, and accounting for multiple levels of attributes and sub-attributes. The study also evaluates the ability of $IVBF_{HSS}$ to manage the multi-dimensional and hierarchical structure of e-learning content, providing a more robust alternative to conventional fuzzy set-based approaches. Through this approach, the research seeks to offer a comprehensive method for generating context-aware, detailed, and highly personalized learning recommendations that reflect the complex preferences of learners.

### Significant contributions

This study provides both theoretical and practical contributions. ${\textbf {Theoretically}}$, it extends hypersoft set theory by incorporating interval-valued bipolar membership, which allows the simultaneous representation of positive and negative preferences as well as uncertainty. ${\textbf {Practically}}$, it improves e-learning recommendation systems by capturing multiple levels of attributes and detailed learner preferences, resulting in more personalized, context-aware, and precise suggestions. By combining advanced set-theoretic concepts with real-world applications, the research offers a robust framework for enhancing the effectiveness of e-learning platforms.

## Preliminaries

In this section, we review some essential concepts related to bipolar hypersoft sets from the literature that are helpful to develop the $IVBF_{HSS}$ Structure.

### **Definition 1**

^3^(Soft set (SS)) Let $\mathbb {O}$ be a universe of discourse, $\mathbb {P}(\mathbb {O})$ the power set of $\mathbb {O}$ and E a set of attributes. Then, the pair $(\mathbb {F}, E),$ where $\mathbb {F}: E \longrightarrow \mathbb {P}(\mathbb {O})$, is called a SS on $\mathbb {O}$.

### **Definition 2**

^32^(Hypersoft set (HSS)) Let $\mathbb {O}$ be a universe of discourse and $\mathbb {P}(\mathbb {O})$ the power set of $\mathbb {O}$. Let $E =\{ \textbf{f}_1, \textbf{f}_2, \textbf{f}_3, \textbf{f}_4,\dots ,\textbf{f}_n\}$ be a set consisting of n disjoint parameters whose corresponding attribute values are $\textbf{G}_1, \textbf{G}_2, \textbf{G}_3, \textbf{G}_4, \dots ,\textbf{G}_n$. Take $\mathbb {G}= \textbf{G}_1 \times \textbf{G}_2 \times \textbf{G}_3 \times \dots \times \textbf{G}_n$, with $\textbf{G}_r \cap \textbf{G}_s = \emptyset$, $r \ne s$, and r, s $\in$ $\{1, 2,\dots , n\}$. The pair $(\mathbb {F}, \mathbb {G})$, where $\mathbb {F}$: $\mathbb {G}$ $\rightarrow$ $\mathbb {P}(\mathbb {O})$, is called a HSS on $\mathbb {O}$.

### **Definition 3**

^28^(Interval-valued bipolar fuzzy set (IVBFS)) Let $\mathbb {O}$ be a universe of discourse. An IVBFS is defined as follows:

$$\begin{aligned} \mathbb {B}^{\textbf{f}}&= \Big \{ \langle {\mathfrak {g}}, {\Bbbk }({\mathfrak {g}}), {\mathbb {H}}({\mathfrak {g}}) \rangle |{\mathfrak {g}} \in \mathbb {O} \Big \} = \Big \{\langle {\mathfrak {g}}, [{\Bbbk }^{{L}^{+}}({\mathfrak {g}}), {\Bbbk }^{{R}^{+}}({\mathfrak {g}})], [{\Bbbk }^{{L}^{-}}({\mathfrak {g}}), {\Bbbk }^{{R}^{-}}({\mathfrak {g}})] \rangle |{\mathfrak {g}} \in \mathbb {O} \Big \}, \end{aligned}$$

where the degree of positive membership function is ${\Bbbk }({\mathfrak {g}}) \subset [0, 1]$ and degree of negative membership function is ${\mathbb {H}}({\mathfrak {g}}) \subset [-1, 0]$, and $\textbf{b}^{\textbf{f}} = \{[{\Bbbk }^{{L}^{+}}, {\Bbbk }^{{R}^{+}}], [{\Bbbk }^{{L}^{-}}, {\Bbbk }^{{R}^{-}}]\}$ is an IVBF number.

Some basic operations on IVBFS are expressed as follows.

### **Lemma 1**

^28^*Let* $\textbf{b}^{\textbf{f}} _{1}$ *and* $\textbf{b}^{\textbf{f}} _{2}$ *be two IVBF numbers, then*

(i) $$\textbf{b}^{\textbf{f}} _{1} \subseteq \textbf{b}^{\textbf{f}} _{2}$$ iff $${\Bbbk }^{{L}^{+}}_1 \le {\Bbbk }^{{L}^{+}}_2$$, $${\Bbbk }^{{R}^{+}}_1 \le {\Bbbk }^{{R}^{+}}_2$$, and $${\mathbb {H}}^{{L}^{-}}_1 \ge {\mathbb {H}}^{{L}^{-}}_2$$, $${\mathbb {H}}^{{L}^{-}}_1 \ge {\mathbb {H}}^{{L}^{-}}_2$$;
(ii) $$\textbf{b}^{\textbf{f}} _{1} \cup \textbf{b}^{\textbf{f}} _{2}$$ = $$([\ max \ \{{\Bbbk }^{{L}^{+}}_1, {\Bbbk }^{{L}^{+}}_2\}$$, $$\ max \{{\Bbbk }^{{R}^{+}}_1, {\Bbbk }^{{R}^{+}}_2\}]$$, $$[ min\ \{{\mathbb {H}}^{{L}^{-}}_1,{\mathbb {H}}^{{L}^{-}}_2\}$$, $$min \ \{{\mathbb {H}}^{{L}^{-}}_1, {\mathbb {H}}^{{L}^{-}}_2$$}]);
(iii) $$\textbf{b}^{\textbf{f}} _{1} \cap \textbf{b}^{\textbf{f}} _{2}$$ = $$([\ min \ \{{\Bbbk }^{{L}^{+}}_1, {\Bbbk }^{{L}^{+}}_2\}$$, $$\ min \{{\Bbbk }^{{R}^{+}}_1, {\Bbbk }^{{R}^{+}}_2\}]$$, $$[ max\ \{{\mathbb {H}}^{{L}^{-}}_1,{\mathbb {H}}^{{L}^{-}}_2\}$$, $$max \ \{{\mathbb {H}}^{{L}^{-}}_1, {\mathbb {H}}^{{L}^{-}}_2$$}]);
(iv) $$({\textbf{b}^{\textbf{f}}})^{'} = \{[1 - {\Bbbk }^{{R}^{+}}, 1- {\Bbbk }^{{L}^{+}}], [|{\Bbbk }^{{R}^{-}}| -1, |{\Bbbk }^{{L}^{-}}| -1]\}$$.

### **Definition 4**

^48^(Bipolar hypersoft set (BHSS)) Assume that $\mathbb {O}$, $\mathbb {P}(\mathbb {O})$, $E =\{ \textbf{f}_1, \textbf{f}_2, \textbf{f}_3, \textbf{f}_4,\dots ,\textbf{f}_n\}$, and $\mathbb {G}= \textbf{G}_1 \times \textbf{G}_2 \times \textbf{G}_3 \times \dots \times \textbf{G}_n$ are the same notions given in Definition 2. The triple $({\Bbbk }, {\mathbb {H}},\mathbb {G})$ is said to be BHSS on $\mathbb {O}$, where ${\Bbbk }$ and ${\mathbb {H}}$ are functions given by ${\Bbbk }: \mathbb {G} \rightarrow \mathbb {P}(\mathbb {O})$ and ${\mathbb {H}}: \mathbb {G} \rightarrow \mathbb {P}(\mathbb {O})$ such that ${\Bbbk }({{\mathbb {j}}_{s}}) \cap {\mathbb {H}}({{\mathbb {j}}_{s}}) = \emptyset$ for all ${{\mathbb {j}}_{s}} \in \mathbb {G}$. A BHSS can be represented as:

$$\begin{aligned} ({\Bbbk }, {\mathbb {H}},\mathbb {G}) = \{({{\mathbb {j}}_{s}}, {\Bbbk }({{\mathbb {j}}_{s}}), {\mathbb {H}}({{\mathbb {j}}_{s}})): {{\mathbb {j}}_{s}} \in \mathbb {G} \wedge {\Bbbk }({{\mathbb {j}}_{s}}) \cap {\mathbb {H}}({{\mathbb {j}}_{s}}) = \emptyset \}. \end{aligned}$$

### **Definition 5**

^48^Assume that $({\Bbbk }_1,{\mathbb {H}}_1,\mathbb {G}_1)$ and $({\Bbbk }_2, {\mathbb {H}}_2,\mathbb {G}_2)$ are two BHSSs. $({\Bbbk }_1, {\mathbb {H}}_1,\mathbb {G}_1)$ is subset of $({\Bbbk }_2, {\mathbb {H}}_2,\mathbb {G}_2)$ if

(i) $$\mathbb {G}_1 \subseteq \mathbb {G}_2$$,
(ii) ${\Bbbk }_1({{\mathbb {j}}_{s}}) \subseteq {\Bbbk }_2({{\mathbb {j}}_{s}})$ and ${\mathbb {H}}_2({{\mathbb {j}}_{s}}) \subseteq {\mathbb {H}}_1({{\mathbb {j}}_{s}})$ for all ${{\mathbb {j}}_{s}} \in \mathbb {G}_1, \mathbb {G}_2.$

### **Definition 6**

^48^The union of two BHSSs $({\Bbbk }_1, {\mathbb {H}}_1,\mathbb {G}_1)$ and $({\Bbbk }_2, {\mathbb {H}}_2,\mathbb {G}_2)$ is $({\Bbbk }_3, {\mathbb {H}}_3, \mathbb {G}_3)$, where $\mathbb {G}_3 = \mathbb {G}_1 \cap \mathbb {G}_2$ and for all ${{\mathbb {j}}_{s}} \in \mathbb {G}_3$,

$$\begin{aligned} {\Bbbk }_3(\ddot{{{\mathbb {j}}_{s}})}= \left\{ \begin{array}{l} {\Bbbk }_1({{\mathbb {j}}_{s}}), \ \ \ if \ {{\mathbb {j}}_{s}} \in \mathbb {G}_1 - \mathbb {G}_2 \\ {\Bbbk }_2({{\mathbb {j}}_{s}}), \ \ \ if \ {{\mathbb {j}}_{s}} \in \mathbb {G}_2 - \mathbb {G}_1 \\ {\Bbbk }_1({{\mathbb {j}}_{s}}) \cup {\Bbbk }_2 {{\mathbb {j}}_{s}} \ \ \ if \ ({{\mathbb {j}}_{s}}) \in \mathbb {G}_1 \cap \mathbb {G}_2 \\ \end{array}\right\} , \end{aligned}$$

$$\begin{aligned} {\mathbb {H}}_3({{\mathbb {j}}_{s}})= \left\{ \begin{array}{l} {\mathbb {H}}_1({{\mathbb {j}}_{s}}), \ \ \ if \ {{\mathbb {j}}_{s}} \in \mathbb {G}_1 - \mathbb {G}_2 \\ {\mathbb {H}}_2({{\mathbb {j}}_{s}}), \ \ \ if \ {{\mathbb {j}}_{s}} \in \mathbb {G}_2 - \mathbb {G}_1 \\ {\mathbb {H}}_1({{\mathbb {j}}_{s}}) \cap {\mathbb {H}}_2({{\mathbb {j}}_{s}}) \ \ \ if \ {{\mathbb {j}}_{s}} \in \mathbb {G}_1 \cap \mathbb {G}_2 \\ \end{array}\right\} . \end{aligned}$$

### **Definition 7**

^48^The intersection of two BHSSs $({\Bbbk }_1, {\mathbb {H}}_1,\mathbb {G}_1)$ and $({\Bbbk }_2, {\mathbb {H}}_2,\mathbb {G}_2)$ is a bipolar hypersoft set $({\Bbbk }_3, {\mathbb {H}}_3, \mathbb {G}_3)$, where $\mathbb {G}_3 = \mathbb {G}_1 \cap \mathbb {G}_2$ and for all ${{\mathbb {j}}_{s}} \in \mathbb {G}_3$,

$$\begin{aligned} {\Bbbk }_3({{\mathbb {j}}_{s}})= \left\{ \begin{array}{l} {\Bbbk }_1({{\mathbb {j}}_{s}}), \ \ \ if \ {{\mathbb {j}}_{s}} \in \mathbb {G}_1 - \mathbb {G}_2 \\ {\Bbbk }_2({{\mathbb {j}}_{s}}), \ \ \ if \ {{\mathbb {j}}_{s}} \in \mathbb {G}_2 - \mathbb {G}_1 \\ {\Bbbk }_1({{\mathbb {j}}_{s}}) \cap {\Bbbk }_2({{\mathbb {j}}_{s}}) \ \ \ if \ {{\mathbb {j}}_{s}} \in \mathbb {G}_1 \cap \mathbb {G}_2 \\ \end{array}\right\} , \end{aligned}$$

$$\begin{aligned} {\mathbb {H}}_3( {{\mathbb {j}}_{s}})= \left\{ \begin{array}{l} {\mathbb {H}}_1( {{\mathbb {j}}_{s}}), \ \ \ if \ {{\mathbb {j}}_{s}} \in \mathbb {G}_1 - \mathbb {G}_2 \\ {\mathbb {H}}_2( {{\mathbb {j}}_{s}}), \ \ \ if \ {{\mathbb {j}}_{s}} \in \mathbb {G}_2 - \mathbb {G}_1 \\ {\mathbb {H}}_1( {{\mathbb {j}}_{s}})\cup {\mathbb {H}}_2( {{\mathbb {j}}_{s}}) \ \ \ if \ {{\mathbb {j}}_{s}} \in \mathbb {G}_1 \cap \mathbb {G}_2\\ \end{array}\right\} . \end{aligned}$$

### Interval-valued bipolar fuzzy hypersoft sets

In this section, the concept of $IVBF_{HSS}$ on $\mathbb {O}$ is introduced and analyzed in depth.

#### **Definition 8**

(Interval-valued bipolar fuzzy hypersoft set ($IVBF_{HSS}$))

Let $\mathbb {O}$ be a universal set and $(\mathbb {P}(\mathbb {O}))^{D[0,1]}$ ($(\mathbb {P}(\mathbb {O}))^{D[-1,0]}$) be the collection of all degrees of positive (negative) membership IVBF subsets of $\mathbb {O}$. Let $E =\{ \textbf{f}_1, \textbf{f}_2, \textbf{f}_3, \textbf{f}_4,\dots ,\textbf{f}_n\}$ be a set consisting of *n* disjoint parameters whose corresponding attribute values are $\textbf{G}_1, \textbf{G}_2, \textbf{G}_3, \textbf{G}_4, \dots ,\textbf{G}_n$. Take $\mathbb {G}= \textbf{G}_1 \times \textbf{G}_2 \times \textbf{G}_3 \times \dots \times \textbf{G}_n$. The triple $({\Bbbk }_{r}, {\mathbb {H}}_{r}, \mathbb {G})$ is called an $IVBF_{HSS}$ on $\mathbb {O}$, where the functions are defined as ${\Bbbk }_{r}: \mathbb {G} \rightarrow (\mathbb {P}(\mathbb {O}))^{D[0,1]}$ and ${\mathbb {H}}_{r}: \mathbb {G} \rightarrow (\mathbb {P}(\mathbb {O}))^{D[-1,0]}$. Also, $IVBF_{HSS}$ can be represented as follows:

$$\begin{aligned} ({\Bbbk }_{r}, {\mathbb {H}}_{r}, \mathbb {G}) = ({\Bbbk }_{r}, {\mathbb {H}}_{r})({\mathbb {j}}_{s}) = \end{aligned}$$

$$\begin{aligned} \{\langle {\mathfrak {g}}_{i}, {\Bbbk }_{r}({\mathfrak {g}}_{i}), {\mathbb {H}}_{r}({\mathfrak {g}}_{i}) \rangle : \forall {\mathfrak {g}}_{i} \in \mathbb {O}, \wedge \ {\Bbbk }_{r}({\mathfrak {g}}_{i}) \cap {\mathbb {H}}_{r}({\mathfrak {g}}_{i}) = \emptyset \}, \end{aligned}$$

where ${\mathbb {j}}_{s} \ \in \ \mathbb {G}$ and $(r, s, i) \in \{1, 2, 3,\dots , n\}$. Where D[0,1] denotes the domain of positive membership intervals, where all interval-valued degrees lie between 0 and 1. It represents the space of possible positive and neutral preferences, and D[-1,0] denotes the domain of negative membership intervals, where all interval-valued degrees lie between -1 and 0. It represents the space of possible negative and neutral preferences.

To provide a clean understanding of the above concept, an example is provided below.

#### *Example 1*

Hospitals possess the complex machinery that is operated by skilled healthcare professionals. However, given the human element involved, occasional errors are not uncommon. In contrast, a highly reputable hospital’s medical staff takes responsibility for any errors and focuses on improving outcomes, while staff in an average hospital tend to shift blame onto others, including administration, procedures, pharmacy, or equipment. Considering numerous factors is critical for identifying the best hospital, as it directly impacts the quality of medical care provided. Therefore, the proposed framework serves as a valuable tool for developing an analysis method tailored to hospitals. By utilizing this structure, healthcare organizations can effectively assess and enhance their overall performance. Let $\mathbb {O}$ = $\{{\mathfrak {g}}_1, {\mathfrak {g}}_2, {\mathfrak {g}}_3, {\mathfrak {g}}_4\}$ be four hospitals, $E = \{\textbf{f}_1, \textbf{f}_2, \textbf{f}_3\}$ be set of parameters where each $\textbf{f}_i$ (i = 1,2,3) stands for the basic amenities, equipment and labs and medical facility, whose corresponding attribute values are $\{\textbf{G}_1, \textbf{G}_2, \textbf{G}_3\}$, respectively. Let

$\textbf{G}_1 = \{ b_{11}$ = easy transport routes, $b_{12}$ = safe food vendors, $b_{13}$ = pharmacy shops, $b_{14}$ = ATM and banking services$\}$,

$\textbf{G}_2 = \{ b_{21}$ = diagnostic laboratories, $b_{22}$ = radio diagnostic equipment, $b_{23}$ = life support equipment $\}$,

$\textbf{G}_3 = \{ b_{31}$ = doctors, $b_{32}$ = nurses, $b_{33}$ = auxiliary workers $\}$.

There are thirty-six possible cases that are to be explored, but for easy computation and best explanation of the performance of the suggested model, only four cases are explored:

$$\begin{aligned} \mathbb {G} = \left\{ \begin{array}{l}\ {\mathbb {j}}_1 = ( b_{11}, b_{21},b_{31}),\ \ \ {\mathbb {j}}_2 = (b_{12}, b_{22}, b_{32}),\\ {\mathbb {j}}_3 = (b_{12}, b_{21},b_{31}),\ \ \ {\mathbb {j}}_4 = (b_{14},b_{23}, b_{33}) \\ \end{array}\right\} . \end{aligned}$$

The positive membership degree of information of $IVBF_{HSS}$ is given as:

$$\begin{aligned} ({\Bbbk }_{r}, \mathbb {G})= \left\{ \begin{array}{l}\ {\Bbbk }({\mathbb {j}}_1)=\{\langle {\mathfrak {g}}_1, [0.5,0.7]\rangle , \langle {\mathfrak {g}}_2, [0.2, 0.3] \rangle ,\\ \langle {\mathfrak {g}}_3, [0.3, 0.5]\rangle , \langle {\mathfrak {g}}_4, [0.1, 0.4]\rangle \},\\ \\ {\Bbbk }({\mathbb {j}}_2)=\{\langle {\mathfrak {g}}_1, [0.2, 0.5] \rangle , \langle {\mathfrak {g}}_2, [0.3, 0.4]\rangle ,\\ \langle {\mathfrak {g}}_3, [0.1, 0.6]\rangle , \langle {\mathfrak {g}}_4, [0.3, 0.9]\rangle \}, \\ \\ {\Bbbk }({\mathbb {j}}_3)=\{ \langle {\mathfrak {g}}_1, [0.5, 0.6]\rangle , \langle {\mathfrak {g}}_2, [0.4, 0.8]\rangle ,\\ \langle {\mathfrak {g}}_3, [0.1, 0.3]\rangle , \langle {\mathfrak {g}}_4, [0.3, 0.7]\rangle \},\\ \\ {\Bbbk }({\mathbb {j}}_4)=\{\langle {\mathfrak {g}}_1, [0.2, 0.3]\rangle , \langle {\mathfrak {g}}_2, [0.3, 0.5]\rangle ,\\ \langle {\mathfrak {g}}_3, [0.5, 0.7]\rangle , \langle {\mathfrak {g}}_4, [0.4, 0.6]\rangle \}\\ \end{array}\right\} . \end{aligned}$$

Also, the negative membership degree of information of $IVBF_{HSS}$ is given as:

$$\begin{aligned} ({\mathbb {H}}_{r}, \mathbb {G})= \left\{ \begin{array}{l}\ {\mathbb {H}}({\mathbb {j}}_1)=\\ \{\langle {\mathfrak {g}}_1, [-0.8,-0.3] \rangle , \langle {\mathfrak {g}}_2, [-0.4, -0.1] \rangle , \\ \langle {\mathfrak {g}}_3, [-0.6, -0.2]\rangle , \langle {\mathfrak {g}}_4, [-0.7, -0.3]\rangle \},\\ {\mathbb {H}}({\mathbb {j}}_2)=\\ \{\langle {\mathfrak {g}}_1, [-0.4, -0.3] \rangle , \langle {\mathfrak {g}}_2, [-0.9, -0.7]\rangle , \\ \langle {\mathfrak {g}}_3, [-0.8, -0.5]\rangle , \langle {\mathfrak {g}}_4, [-0.5, -0.4]\rangle \}, \\ {\mathbb {H}}({\mathbb {j}}_3)=\\ \{ \langle {\mathfrak {g}}_1, [-0.3, -0.1] \rangle , \langle {\mathfrak {g}}_2, [-0.5, -0.2]\rangle ,\\ \langle {\mathfrak {g}}_3, [-0.7, -0.5]\rangle , \langle {\mathfrak {g}}_4, [-0.4, -0.2]\rangle \},\\ {\mathbb {H}}({\mathbb {j}}_4)=\\ \{\langle {\mathfrak {g}}_1, [-0.6, -0.4]\rangle , \langle {\mathfrak {g}}_2, [-0.4, -0.1]\rangle ,\\ \langle {\mathfrak {g}}_3, [-0.8, -0.6]\rangle , \langle {\mathfrak {g}}_4, [-0.5, -0.2]\rangle \},\\ \end{array}\right\} . \end{aligned}$$

The $IVBF_{HSS}$ takes the form:

$$\begin{aligned} ({\Bbbk }_{r}, {\mathbb {H}}_{r}, \mathbb {G})= \left\{ \begin{array}{l}\ ({\Bbbk }_{r}, {\mathbb {H}}_{r})({\mathbb {j}}_1)=\{(\langle {\mathfrak {g}}_1, [0.5,0.7], [-0.8,-0.3] \rangle , \langle {\mathfrak {g}}_2, [0.2, 0.3], [-0.4, -0.1] \rangle ,\\ \langle {\mathfrak {g}}_3, [0.3, 0.5], [-0.6, -0.2]\rangle , \langle {\mathfrak {g}}_4, [0.1, 0.4], [-0.7, -0.3]\rangle ),\\ ({\Bbbk }_{r}, {\mathbb {H}}_{r})({\mathbb {j}}_2)=\{(\langle {\mathfrak {g}}_1, [0.2, 0.5], [-0.4, -0.3] \rangle , \langle {\mathfrak {g}}_2, [0.3, 0.4], [-0.9, -0.7]\rangle , \\ \langle {\mathfrak {g}}_3, [0.1, 0.6], [-0.8, -0.5]\rangle , \langle {\mathfrak {g}}_4, [0.3, 0.9], [-0.5, -0.4]\rangle ),\\ ({\Bbbk }_{r}, {\mathbb {H}}_{r})({\mathbb {j}}_3)=\{ (\langle {\mathfrak {g}}_1, [0.5, 0.6], [-0.3, -0.1] \rangle , \langle {\mathfrak {g}}_2, [0.4, 0.8], [-0.5, -0.2]\rangle ,\\ \langle {\mathfrak {g}}_3, [0.1, 0.3], [-0.7, -0.5]\rangle , \langle {\mathfrak {g}}_4, [0.3, 0.7], [-0.4, -0.2]\rangle )\},\\ ({\Bbbk }_{r}, {\mathbb {H}}_{r})({\mathbb {j}}_4)=\{(\langle {\mathfrak {g}}_1, [0.2, 0.3], [-0.6, -0.4]\rangle , \langle {\mathfrak {g}}_2, [0.3, 0.5], [-0.4, -0.1]\rangle ,\\ \langle {\mathfrak {g}}_3, [0.5, 0.7], [-0.8, -0.6]\rangle , \langle {\mathfrak {g}}_4, [0.4, 0.6], [-0.5, -0.2]\rangle )\}\\ \end{array}\right\} . \end{aligned}$$

$$\square$$

#### **Definition 9**

Let $({\Bbbk }_{1}, {\mathbb {H}}_{1}, \mathbb {G}_1)$ and $({\Bbbk }_{2}, {\mathbb {H}}_{2}, \mathbb {G}_2)$ be two $IVBF_{HSS}$s, then $({\Bbbk }_{1}, {\mathbb {H}}_{1}, \mathbb {G}_1)$ is subset of $({\Bbbk }_{2}, {\mathbb {H}}_{2}, \mathbb {G}_2)$ if

(i) $$\mathbb {G}_1 \subseteq \mathbb {G}_2$$,
(ii) ${\Bbbk }_1({\mathbb {j}}_{s}) \subseteq {\Bbbk }_2({\mathbb {j}}_{s})$ and ${\mathbb {H}}_1({\mathbb {j}}_{s}) \subseteq {\mathbb {H}}_2({\mathbb {j}}_{s})$ for all ${\mathbb {j}}_{s} \in \mathbb {G}_1, \mathbb {G}_2.$

#### *Example 2*

Consider $({\Bbbk }_{1}, {\mathbb {H}}_{1}, \mathbb {G}_1)$ and $({\Bbbk }_{2}, {\mathbb {H}}_{2}, \mathbb {G}_2)$ as two $IVBF_{HSS}s$,:

$$({\Bbbk }_{1}, {\mathbb {H}}_{1}, \mathbb {G}_1)= \left\{ \begin{array}{l}\ ({\Bbbk }_{1}, {\mathbb {H}}_{1})({\mathbb {j}}_1)=\{\langle {\mathfrak {g}}_{1}, [0.4, 0.7], [-0.7, -0.3] \rangle , \langle {\mathfrak {g}}_{2}, [0.1, 0.3], [-0.4, -0.2]\rangle ,\\ \\ \langle {\mathfrak {g}}_{3}, [0.2, 0.5], [-0.6, -0.5]\rangle , \langle {\mathfrak {g}}_{4}, [0.1, 0.4], [-0.7, -0.4]\rangle \}, \\ \\ ({\Bbbk }_{1}, {\mathbb {H}}_{1})({\mathbb {j}}_2)=\{\langle {\mathfrak {g}}_{1}, [0.2, 0.5], [-0.4, -0.3]\rangle , \langle {\mathfrak {g}}_{2}, [0.1, 0.4], [-0.9, -0.7]\rangle , \\ \\ \langle {\mathfrak {g}}_{3}, [0.1, 0.6], [-0.8, -0.5]\rangle , \langle {\mathfrak {g}}_{4}, [0.2, 0.8], [-0.5, -0.4]\rangle \} \end{array}\right\}$$,

and

$$({\Bbbk }_{2}, {\mathbb {H}}_{2}, \mathbb {G}_2)= \left\{ \begin{array}{l}\ ({\Bbbk }_{2}, {\mathbb {H}}_{2})({\mathbb {j}}_1)=\{ (\langle {\mathfrak {g}}_{1}, [0.5, 0.9], [-0.8, -0.2]\rangle , \langle {\mathfrak {g}}_{2}, [0.2, 0.6], [-0.5, -0.1]\rangle , \\ \\ \langle {\mathfrak {g}}_{3}, [0.3, 0.7], [-0.7, -0.4]\rangle , \langle {\mathfrak {g}}_{4}, [0.2, 0.5], [-0.8, -0.3]\rangle ,\\ \\ ({\Bbbk }_{2}, {\mathbb {H}}_{2})({\mathbb {j}}_2)=\{(\langle {\mathfrak {g}}_{1}, [0.3, 0.6], [-0.6, -0.2]\rangle , \langle {\mathfrak {g}}_{2}, [0.3, 0.5], [-0.9, -0.5]\rangle , \\ \\ \langle {\mathfrak {g}}_{3}, [0.2, 0.7], [-0.9, -0.4]\rangle , \langle {\mathfrak {g}}_{4}, [0.3, 0.9], [-0.7, -0.3]\rangle \} \end{array}\right\}$$.

Then, by Definition 9, we get: $$({\Bbbk }_{1}, {\mathbb {H}}_{1}, \mathbb {G}_1) \subseteq ({\Bbbk }_{2}, {\mathbb {H}}_{2}, \mathbb {G}_2).$$$$\square$$

#### **Definition 10**

The complement of the $IVBF_{HSS}$ $({\Bbbk }, {\mathbb {H}}, \mathbb {G})$ is defined by

$$\begin{aligned} ({\Bbbk }, {\mathbb {H}}, \mathbb {G})^{c} = (({\Bbbk })^{c}, ({\mathbb {H}})^{c}, \mathbb {G}). \end{aligned}$$

#### *Example 3*

Assume that $({\Bbbk }, {\mathbb {H}}, \mathbb {G})$ is an arbitrary $IVBF_{HSS}$ with the following structure

$$\begin{aligned} ({\Bbbk }, {\mathbb {H}}, \mathbb {G}) = \left\{ \begin{array}{l}\ ({\Bbbk }, {\mathbb {H}})({\mathbb {j}})=\{ \langle {\mathfrak {g}}_1, [0.6, 0.9], [-0.7, -0.3] \rangle ,\\ \langle {\mathfrak {g}}_2, [0.4, 0.8], [-0.7, -0.4]\rangle , \\ \\ \langle {\mathfrak {g}}_3, [0.6, 0.7], [-0.7, -0.5]\rangle ,\\ \langle {\mathfrak {g}}_4, [0.3, 0.7], [-0.8, -0.5]\rangle ,\} \\ \end{array}\right\} . \end{aligned}$$

Then

$$\begin{aligned} ({\Bbbk }, {\mathbb {H}}, \mathbb {G})^{c}= \left\{ \begin{array}{l}\ ({\Bbbk }, {\mathbb {H}})({\mathbb {j}})=\{\langle {\mathfrak {g}}_1, [0.1, 0.4], [-0.7, -0.3]\rangle ,\\ \langle {\mathfrak {g}}_2, [0.2, 0.6], [-0.6, -0.3]\rangle , \\ \\ \langle {\mathfrak {g}}_3, [0.3, 0.4], [-0.5, -0.3]\rangle ,\\ \langle {\mathfrak {g}}_4, [0.3, 0.7], [-0.5, -0.2]\rangle \}\\ \end{array}\right\} . \end{aligned}$$

$$\square$$

#### **Definition 11**

The extended union of $({\Bbbk }_1, {\mathbb {H}}_1, \mathbb {G}_1)$ and $({\Bbbk }_2, {\mathbb {H}}_2, \mathbb {G}_2)$ is denoted as

$$\begin{aligned} ({\Bbbk }_3, {\mathbb {H}}_3, \mathbb {G}_3)=({\Bbbk }_1, {\mathbb {H}}_1, \mathbb {G}_1) \cup _{e} ({\Bbbk }_2, {\mathbb {H}}_2, \mathbb {G}_2), \end{aligned}$$

where $\mathbb {G}_3 = \mathbb {G}_1 \cup _{e} \mathbb {G}_2$ and for all ${\mathbb {j}} \in \mathbb {G}_3$,

$$\begin{aligned} {\Bbbk }_3({\mathbb {j}}_{s})= \left\{ \begin{array}{l} {\Bbbk }_1({\mathbb {j}}_{s}), \ \ \ if \ {\mathbb {j}}_{s} \in \mathbb {G}_1 - \mathbb {G}_2 \\ {\Bbbk }_2({\mathbb {j}}_{s}), \ \ \ if \ {\mathbb {j}}_{s} \in \mathbb {G}_2 - \mathbb {G}_1 \\ {\Bbbk }_1({\mathbb {j}}_{s}) \cup _{e} {\Bbbk }_2({\mathbb {j}}_{s}) \ \ \ if \ {\mathbb {j}}_{s} \in \mathbb {G}_1 \cap _{e} \mathbb {G}_2 \\ \end{array}\right\} , \end{aligned}$$

$$\begin{aligned} {\mathbb {H}}_3({\mathbb {j}}_{s})= \left\{ \begin{array}{l} {\mathbb {H}}_1({\mathbb {j}}_{s}), \ \ \ if \ {\mathbb {j}}_{s} \in \mathbb {G}_1 - \mathbb {G}_2 \\ {\mathbb {H}}_2({\mathbb {j}}_{s}), \ \ \ if \ {\mathbb {j}}_{s} \in \mathbb {G}_2 - \mathbb {G}_1 \\ {\mathbb {H}}_1( {\mathbb {j}}_{s}) \cap _{e} {\mathbb {H}}_2( {\mathbb {j}}_{s}) \ \ \ if \ {\mathbb {j}}_{s} \in \mathbb {G}_1 \cap _{e} \mathbb {G}_2 \\ \end{array}\right\} . \end{aligned}$$

#### *Example 4*

Considering Example 2, the extended union of $({\Bbbk }_1, {\mathbb {H}}_1, \mathbb {G}_1)$ and $({\Bbbk }_2, {\mathbb {H}}_2, \mathbb {G}_2)$ is computed as:

$$\begin{aligned} ({\Bbbk }_4, {\mathbb {H}}_4, \mathbb {G}_3)=\left\{ \begin{array}{l}\ ({\Bbbk }_{4}, {\mathbb {H}}_{4})({\mathbb {j}}_1)=\{\langle {\mathfrak {g}}_{1}, [0.4, 0.7], [-0.7, -0.3] \rangle , \langle {\mathfrak {g}}_{2}, [0.1, 0.3], [-0.4, -0.2]\rangle ,\\ \\ \langle {\mathfrak {g}}_{3}, [0.2, 0.5], [-0.6, -0.5]\rangle , \langle {\mathfrak {g}}_{4}, [0.1, 0.4], [-0.7, -0.4]\rangle \}, \\ \\ ({\Bbbk }_{4}, {\mathbb {H}}_{4})({\mathbb {j}}_2)=\{\langle {\mathfrak {g}}_{1}, [0.2, 0.5], [-0.4, -0.3]\rangle , \langle {\mathfrak {g}}_{2}, [0.1, 0.4], [-0.9, -0.7]\rangle , \\ \\ \langle {\mathfrak {g}}_{3}, [0.1, 0.6], [-0.8, -0.5]\rangle , \langle {\mathfrak {g}}_{4}, [0.2, 0.8], [-0.5, -0.4]\rangle \}\\ \end{array}\right\} . \end{aligned}$$

$$\square$$

#### **Definition 12**

The extended intersection of $({\Bbbk }_1, {\mathbb {H}}_1, \mathbb {G}_1)$ and $({\Bbbk }_2, {\mathbb {H}}_2, \mathbb {G}_2)$ is denoted by:

$$\begin{aligned} ({\Bbbk }_4, {\mathbb {H}}_4, \mathbb {G}_4)=({\Bbbk }_1, {\mathbb {H}}_1, \mathbb {G}_1) \cap _{e} ({\Bbbk }_2, {\mathbb {H}}_2, \mathbb {G}_2), \end{aligned}$$

where $\mathbb {G}_4 = \mathbb {G}_1 \cup _{e} \mathbb {G}_2$ and for all ${\mathbb {j}}_{s} \in \mathbb {G}_3$,

$$\begin{aligned} {\Bbbk }_4({\mathbb {j}}_{s})= \left\{ \begin{array}{l} {\Bbbk }_1({\mathbb {j}}_{s}), \ \ \ if \ {\mathbb {j}}_{s} \in \mathbb {G}_1 - \mathbb {G}_2 \\ {\Bbbk }_2({\mathbb {j}}_{s}), \ \ \ if \ {\mathbb {j}}_{s} \in \mathbb {G}_2 - \mathbb {G}_1 \\ {\Bbbk }_1({\mathbb {j}}_{s}) \cap _{e} {\Bbbk }_2({\mathbb {j}}_{s}) \ \ \ if \ {\mathbb {j}}_{s} \in \mathbb {G}_1 \cap _{e} \mathbb {G}_2 \\ \end{array}\right\} , \end{aligned}$$

$$\begin{aligned} {\mathbb {H}}_4( {\mathbb {j}}_{s})= \left\{ \begin{array}{l} {\mathbb {H}}_1( {\mathbb {j}}_{s}), \ \ \ if \ {\mathbb {j}}_{s} \in \mathbb {G}_1 - \mathbb {G}_2 \\ {\mathbb {H}}_2( {\mathbb {j}}_{s}), \ \ \ if \ {\mathbb {j}}_{s} \in \mathbb {G}_2 - \mathbb {G}_1 \\ {\mathbb {H}}_1( {\mathbb {j}}_{s}) \cup _{e} {\mathbb {H}}_2({\mathbb {j}}_{s}) \ \ \ if \ {\mathbb {j}}_{s} \in \mathbb {G}_1 \cap _{e} \mathbb {G}_2 \\ \end{array}\right\} . \end{aligned}$$

#### *Example 5*

Considering Example 2, the extended intersection of $({\Bbbk }_1, {\mathbb {H}}_1, \mathbb {G}_1)$ and $({\Bbbk }_2, {\mathbb {H}}_2, \mathbb {G}_2)$ is computed by:

$$\begin{aligned} ({\Bbbk }_4, {\mathbb {H}}_4, \mathbb {G}_3)= \left\{ \begin{array}{l}\ ({\Bbbk }_{4}, {\mathbb {H}}_{4})({\mathbb {j}}_1)=\{\langle {\mathfrak {g}}_{1}, [0.4, 0.7], [-0.7, -0.3] \rangle , \langle {\mathfrak {g}}_{2}, [0.1, 0.3], [-0.4, -0.2]\rangle ,\\ \\ \langle {\mathfrak {g}}_{3}, [0.2, 0.5], [-0.6, -0.5]\rangle , \langle {\mathfrak {g}}_{4}, [0.1, 0.4], [-0.7, -0.4]\rangle \}, \\ ({\Bbbk }_{4}, {\mathbb {H}}_{4})({\mathbb {j}}_2)=\{\langle {\mathfrak {g}}_{1}, [0.2, 0.5], [-0.4, -0.3]\rangle , \langle {\mathfrak {g}}_{2}, [0.1, 0.4], [-0.9, -0.7]\rangle , \\ \\ \langle {\mathfrak {g}}_{3}, [0.1, 0.6], [-0.8, -0.5]\rangle , \langle {\mathfrak {g}}_{4}, [0.2, 0.8], [-0.5, -0.4]\rangle \}\\ \end{array}\right\} . \end{aligned}$$

$$\square$$

#### **Proposition 1**

*Let* $({\Bbbk }, {\mathbb {H}}, \mathbb {G})$ *be an* $IVBF_{HSS}$ *on* $\mathbb {O}$. *Then, *

(i) $$({\Bbbk }, {\mathbb {H}}, \mathbb {G}) \cup _{e} ({\Bbbk }, {\mathbb {H}}, \mathbb {G})$$ = $$({\Bbbk }, {\mathbb {H}}, \mathbb {G})$$,
(ii) $$({\Bbbk }, {\mathbb {H}}, \mathbb {G}) \cap _{e}({\Bbbk }, {\mathbb {H}}, \mathbb {G})$$ = $$({\Bbbk }, {\mathbb {H}}, \mathbb {G})$$.

#### *Proof*

(i) We know that $({\Bbbk }_3, {\mathbb {H}}_3, \mathbb {G}_3)$ = $({\Bbbk }, {\mathbb {H}}, \mathbb {G} ) \cup _{e} ({\Bbbk }, {\mathbb {H}}, \mathbb {G} )$, where $\mathbb {G}_3 = \mathbb {G} \cup _{e} \mathbb {G}$ and for all ${\mathbb {j}} \in \mathbb {G}_3$,

$$\begin{aligned}{\Bbbk }_3({\mathbb {j}}_{s})= \left\{ \begin{array}{l} {\Bbbk } ({\mathbb {j}}_{s}), \ \ \ if \ {\mathbb {j}}_{s} \in \mathbb {G} - \mathbb {G} \\ {\Bbbk } ({\mathbb {j}}_{s}), \ \ \ if \ {\mathbb {j}}_{s} \in \mathbb {G} - \mathbb {G} \\ {\Bbbk } ({\mathbb {j}}_{s}) \cup _{e} {\Bbbk } ({\mathbb {j}}_{s}) \ \ \ if \ {\mathbb {j}}_{s} \in \mathbb {G} \cap _{e} \mathbb {G} \\ \end{array}\right\} , \end{aligned}$$

$$\begin{aligned}{\mathbb {H}}_3({\mathbb {j}}_{s})= \left\{ \begin{array}{l} {\mathbb {H}} ({\mathbb {j}}_{s}), \ \ \ if \ {\mathbb {j}}_{s} \in \mathbb {G} - \mathbb {G} \\ {\mathbb {H}} ({\mathbb {j}}_{s}), \ \ \ if \ {\mathbb {j}}_{s} \in \mathbb {G} - \mathbb {G} \\ {\mathbb {H}} ( {\mathbb {j}}_{s}) \ \ \ if \ {\mathbb {j}}_{s} \in \mathbb {G} \\ \end{array}\right\} , \end{aligned}$$

and also

$$\begin{aligned}{\Bbbk }_3({\mathbb {j}}_{s})= \left\{ \begin{array}{l} {\Bbbk } ({\mathbb {j}}_{s}), \ \ \ if \ {\mathbb {j}}_{s} \in \emptyset \\ {\Bbbk } ({\mathbb {j}}_{s}), \ \ \ if \ {\mathbb {j}}_{s} \in \emptyset \\ {\Bbbk } ({\mathbb {j}}_{s}) \ \ \ if \ {\mathbb {j}}_{s} \in \mathbb {G} \\ \end{array}\right\} , \end{aligned}$$

$$\begin{aligned}{\mathbb {H}}_3({\mathbb {j}}_{s})= \left\{ \begin{array}{l} {\mathbb {H}} ({\mathbb {j}}_{s}), \ \ \ if \ {\mathbb {j}}_{s} \in \emptyset \\ {\mathbb {H}} ({\mathbb {j}}_{s}), \ \ \ if \ {\mathbb {j}}_{s} \in \emptyset \\ {\mathbb {H}} ( {\mathbb {j}}_{s}) \ \ \ if \ {\mathbb {j}}_{s} \in \mathbb {G} \\ \end{array}\right\} . \end{aligned}$$

Hence, it is proved that $({\Bbbk }, {\mathbb {H}}, \mathbb {G}) \cup _{e} ({\Bbbk }, {\mathbb {H}}, \mathbb {G})$ = $({\Bbbk }, {\mathbb {H}}, \mathbb {G})$.

(ii) We know that $({\Bbbk }_3, {\mathbb {H}}_3, \mathbb {G}_3)$ = $({\Bbbk }, {\mathbb {H}}, \mathbb {G} ) \cap _{e} ({\Bbbk }, {\mathbb {H}}, \mathbb {G} )$, where $\mathbb {G}_3 = \mathbb {G} \cup _{e} \mathbb {G}$ and for all ${\mathbb {j}} \in \mathbb {G}_3$,

$$\begin{aligned} {\Bbbk }_3({\mathbb {j}}_{s})= \left\{ \begin{array}{l} {\Bbbk } ({\mathbb {j}}_{s}), \ \ \ if \ {\mathbb {j}}_{s} \in \mathbb {G} - \mathbb {G} \\ {\Bbbk } ({\mathbb {j}}_{s}), \ \ \ if \ {\mathbb {j}}_{s} \in \mathbb {G} - \mathbb {G} \\ {\Bbbk } ({\mathbb {j}}_{s}) \cap _{e} {\Bbbk } ({\mathbb {j}}_{s}) \ \ \ if \ {\mathbb {j}}_{s} \in \mathbb {G} \cap _{e} \mathbb {G} \\ \end{array}\right\} , \end{aligned}$$

$$\begin{aligned} {\mathbb {H}}_3({\mathbb {j}}_{s})= \left\{ \begin{array}{l} {\mathbb {H}} ({\mathbb {j}}_{s}), \ \ \ if \ {\mathbb {j}}_{s} \in \mathbb {G} - \mathbb {G} \\ {\mathbb {H}} ({\mathbb {j}}_{s}), \ \ \ if \ {\mathbb {j}}_{s} \in \mathbb {G} - \mathbb {G} \\ {\mathbb {H}} ( {\mathbb {j}}_{s}) \cup _{e} {\mathbb {H}} ( {\mathbb {j}}_{s}) \ \ \ if \ {\mathbb {j}}_{s} \in \mathbb {G} \cap _{e} \mathbb {G} \\ \end{array}\right\} , \end{aligned}$$

and also,

$$\begin{aligned} {\Bbbk }_3({\mathbb {j}}_{s})= \left\{ \begin{array}{l} {\Bbbk } ({\mathbb {j}}_{s}), \ \ \ if \ {\mathbb {j}}_{s} \in \emptyset \\ {\Bbbk } ({\mathbb {j}}_{s}), \ \ \ if \ {\mathbb {j}}_{s} \in \emptyset \\ {\Bbbk } ({\mathbb {j}}_{s}) \ \ \ if \ {\mathbb {j}}_{s} \in \mathbb {G} \\ \end{array}\right\} , \end{aligned}$$

$$\begin{aligned} {\mathbb {H}}_3({\mathbb {j}}_{s})= \left\{ \begin{array}{l} {\mathbb {H}} ({\mathbb {j}}_{s}), \ \ \ if \ {\mathbb {j}}_{s} \in \emptyset \\ {\mathbb {H}} ({\mathbb {j}}_{s}), \ \ \ if \ {\mathbb {j}}_{s} \in \emptyset \\ {\mathbb {H}} ( {\mathbb {j}}_{s}) \ \ \ if \ {\mathbb {j}}_{s} \in \mathbb {G} \\ \end{array}\right\} . \end{aligned}$$

Hence, it is proved that $({\Bbbk }, {\mathbb {H}}, \mathbb {G}) \cap _{e} ({\Bbbk }, {\mathbb {H}}, \mathbb {G})$ = $({\Bbbk }, {\mathbb {H}}, \mathbb {G})$. $\square$

#### **Theorem 2**

*(Commutative property) Let* $({\Bbbk }_1, {\mathbb {H}}_1, \mathbb {G}_1)$ *and* $({\Bbbk }_2, {\mathbb {H}}_2, \mathbb {G}_2)$ *be two* $IVBF_{HSS}s$ *on* $\mathbb {O}$. *Then,*

(i) $$({\Bbbk }_1, {\mathbb {H}}_1, \mathbb {G}_1) \cup _{e} ({\Bbbk }_2, {\mathbb {H}}_2, \mathbb {G}_2)$$ = $$({\Bbbk }_2, {\mathbb {H}}_2, \mathbb {G}_2) \cup _{e} ({\Bbbk }_1, {\mathbb {H}}_1, \mathbb {G}_1)$$,
(ii) $$({\Bbbk }_1, {\mathbb {H}}_1, \mathbb {G}_1) \cap _{e} ({\Bbbk }_2, {\mathbb {H}}_2, \mathbb {G}_2)$$ = $$({\Bbbk }_2, {\mathbb {H}}_2, \mathbb {G}_2) \cap _{e} ({\Bbbk }_1, {\mathbb {H}}_1, \mathbb {G}_1)$$.

#### *Proof*

(i) We know that the extended union of $({\Bbbk }_1, {\mathbb {H}}_1, \mathbb {G}_1)$ and $({\Bbbk }_2, {\mathbb {H}}_2, \mathbb {G}_2)$ is represented by

$$\begin{aligned} ({\Bbbk }_3, {\mathbb {H}}_3, \mathbb {G}_3)=({\Bbbk }_1, {\mathbb {H}}_1, \mathbb {G}_1) \cup _{e} ({\Bbbk }_2, {\mathbb {H}}_2, \mathbb {G}_2), \end{aligned}$$

where $\mathbb {G}_3 = \mathbb {G}_1 \cup _{e} \mathbb {G}_2$ and for all ${\mathbb {j}} \in \mathbb {G}_3$,

$$\begin{aligned}{\Bbbk }_3({\mathbb {j}}_{s})= \left\{ \begin{array}{l} {\Bbbk }_1({\mathbb {j}}_{s}), \ \ \ if \ {\mathbb {j}}_{s} \in \mathbb {G}_1 - \mathbb {G}_2 \\ {\Bbbk }_2({\mathbb {j}}_{s}), \ \ \ if \ {\mathbb {j}}_{s} \in \mathbb {G}_2 - \mathbb {G}_1 \\ {\Bbbk }_1({\mathbb {j}}_{s}) \cup _{e} {\Bbbk }_2({\mathbb {j}}_{s}) \ \ \ if \ {\mathbb {j}}_{s} \in \mathbb {G}_1 \cap _{e} \mathbb {G}_2 \\ \end{array}\right\} , \end{aligned}$$

$$\begin{aligned} {\mathbb {H}}_3({\mathbb {j}}_{s})= \left\{ \begin{array}{l} {\mathbb {H}}_1({\mathbb {j}}_{s}), \ \ \ if \ {\mathbb {j}}_{s} \in \mathbb {G}_1 - \mathbb {G}_2 \\ {\mathbb {H}}_2({\mathbb {j}}_{s}), \ \ \ if \ {\mathbb {j}}_{s} \in \mathbb {G}_2 - \mathbb {G}_1 \\ {\mathbb {H}}_1( {\mathbb {j}}_{s}) \cap _{e} {\mathbb {H}}_2( {\mathbb {j}}_{s}) \ \ \ if \ {\mathbb {j}}_{s} \in \mathbb {G}_1 \cap _{e} \mathbb {G}_2 \\ \end{array}\right\} , \end{aligned}$$

and also,

$$\begin{aligned} {\Bbbk }_3({\mathbb {j}}_{s})= \left\{ \begin{array}{l} {\Bbbk }_2({\mathbb {j}}_{s}), \ \ \ if \ {\mathbb {j}}_{s} \in \mathbb {G}_2 - \mathbb {G}_1 \\ {\Bbbk }_1({\mathbb {j}}_{s}), \ \ \ if \ {\mathbb {j}}_{s} \in \mathbb {G}_1 - \mathbb {G}_2 \\ {\Bbbk }_2({\mathbb {j}}_{s}) \cup _{e} {\Bbbk }_1({\mathbb {j}}_{s}) \ \ \ if \ {\mathbb {j}}_{s} \in \mathbb {G}_2 \cap _{e} \mathbb {G}_1 \\ \end{array}\right\} , \end{aligned}$$

$$\begin{aligned} {\mathbb {H}}_3({\mathbb {j}}_{s})= \left\{ \begin{array}{l} {\mathbb {H}}_2({\mathbb {j}}_{s}), \ \ \ if \ {\mathbb {j}}_{s} \in \mathbb {G}_2 - \mathbb {G}_1 \\ {\mathbb {H}}_1({\mathbb {j}}_{s}), \ \ \ if \ {\mathbb {j}}_{s} \in \mathbb {G}_1 - \mathbb {G}_2 \\ {\mathbb {H}}_2( {\mathbb {j}}_{s}) \cap _{e} {\mathbb {H}}_1( {\mathbb {j}}_{s}) \ \ \ if \ {\mathbb {j}}_{s} \in \mathbb {G}_2 \cap _{e} \mathbb {G}_1 \\ \end{array}\right\} . \end{aligned}$$

This implies

$$\begin{aligned} ({\Bbbk }_3, {\mathbb {H}}_3, \mathbb {G}_3)=({\Bbbk }_2, {\mathbb {H}}_2, \mathbb {G}_2) \cup _{e} ({\Bbbk }_1, {\mathbb {H}}_1, \mathbb {G}_1). \end{aligned}$$

Hence, $$({\Bbbk }_1, {\mathbb {H}}_1, \mathbb {G}_1) \cup _{e} ({\Bbbk }_2, {\mathbb {H}}_2, \mathbb {G}_1)$$ = $$({\Bbbk }_2, {\mathbb {H}}_2, \mathbb {G}_2) \cup _{e} ({\Bbbk }_1, {\mathbb {H}}_1, \mathbb {G}_1)$$.

(ii) We know that the extended intersection of $({\Bbbk }_1, {\mathbb {H}}_1, \mathbb {G}_1)$ and $({\Bbbk }_2, {\mathbb {H}}_2, \mathbb {G}_2)$ is represented by

$$\begin{aligned} ({\Bbbk }_3, {\mathbb {H}}_3, \mathbb {G}_3) = ({\Bbbk }_1, {\mathbb {H}}_1, \mathbb {G}_1) \cap _{e} ({\Bbbk }_2, {\mathbb {H}}_2, \mathbb {G}_2), \end{aligned}$$

where $\mathbb {G}_3 = \mathbb {G}_1 \cup _{e} \mathbb {G}_2$ and for all ${\mathbb {j}} \in \mathbb {G}_3$,

$$\begin{aligned} {\Bbbk }_3({\mathbb {j}}_{s})= \left\{ \begin{array}{l} {\Bbbk }_1({\mathbb {j}}_{s}), \ \ \ if \ {\mathbb {j}}_{s} \in \mathbb {G}_1 - \mathbb {G}_2 \\ {\Bbbk }_2({\mathbb {j}}_{s}), \ \ \ if \ {\mathbb {j}}_{s} \in \mathbb {G}_2 - \mathbb {G}_1 \\ {\Bbbk }_1({\mathbb {j}}_{s}) \cap _{e} {\Bbbk }_2({\mathbb {j}}_{s}) \ \ \ if \ {\mathbb {j}}_{s} \in \mathbb {G}_1 \cap _{e} \mathbb {G}_2 \\ \end{array}\right\} , \end{aligned}$$

$$\begin{aligned} {\mathbb {H}}_3({\mathbb {j}}_{s})= \left\{ \begin{array}{l} {\mathbb {H}}_1({\mathbb {j}}_{s}), \ \ \ if \ {\mathbb {j}}_{s} \in \mathbb {G}_1 - \mathbb {G}_2 \\ {\mathbb {H}}_2({\mathbb {j}}_{s}), \ \ \ if \ {\mathbb {j}}_{s} \in \mathbb {G}_2 - \mathbb {G}_1 \\ {\mathbb {H}}_1( {\mathbb {j}}_{s}) \cup _{e} {\mathbb {H}}_2( {\mathbb {j}}_{s}) \ \ \ if \ {\mathbb {j}}_{s} \in \mathbb {G}_1 \cap _{e} \mathbb {G}_2 \\ \end{array}\right\} , \end{aligned}$$

and also,

$$\begin{aligned} {\Bbbk }_3({\mathbb {j}}_{s})= \left\{ \begin{array}{l} {\Bbbk }_2({\mathbb {j}}_{s}), \ \ \ if \ {\mathbb {j}}_{s} \in \mathbb {G}_2 - \mathbb {G}_1 \\ {\Bbbk }_1({\mathbb {j}}_{s}), \ \ \ if \ {\mathbb {j}}_{s} \in \mathbb {G}_1 - \mathbb {G}_2 \\ {\Bbbk }_2({\mathbb {j}}_{s}) \cap _{e} {\Bbbk }_1({\mathbb {j}}_{s}) \ \ \ if \ {\mathbb {j}}_{s} \in \mathbb {G}_2 \cap _{e} \mathbb {G}_1 \\ \end{array}\right\} , \end{aligned}$$

$$\begin{aligned} {\mathbb {H}}_3({\mathbb {j}}_{s})= \left\{ \begin{array}{l} {\mathbb {H}}_2({\mathbb {j}}_{s}), \ \ \ if \ {\mathbb {j}}_{s} \in \mathbb {G}_2 - \mathbb {G}_1 \\ {\mathbb {H}}_1({\mathbb {j}}_{s}), \ \ \ if \ {\mathbb {j}}_{s} \in \mathbb {G}_1 - \mathbb {G}_2 \\ {\mathbb {H}}_2( {\mathbb {j}}_{s}) \cup _{e} {\mathbb {H}}_1( {\mathbb {j}}_{s}) \ \ \ if \ {\mathbb {j}}_{s} \in \mathbb {G}_2 \cap _{e} \mathbb {G}_1 \\ \end{array}\right\} . \end{aligned}$$

This implies

$$\begin{aligned} ({\Bbbk }_3, {\mathbb {H}}_3, \mathbb {G}_3)=({\Bbbk }_2, {\mathbb {H}}_2, \mathbb {G}_2) \cap _{e} ({\Bbbk }_1, {\mathbb {H}}_1, \mathbb {G}_1).\end{aligned}$$

Hence, $({\Bbbk }_1, {\mathbb {H}}_1, \mathbb {G}_1) \cap _{e} ({\Bbbk }_2, {\mathbb {H}}_2, \mathbb {G}_1)$ = $({\Bbbk }_2, {\mathbb {H}}_2, \mathbb {G}_2) \cap _{e} ({\Bbbk }_1, {\mathbb {H}}_1, \mathbb {G}_1)$, and this ends the proof. $\square$

#### **Theorem 3**

*(Associative property) Let* $({\Bbbk }_1, {\mathbb {H}}_1, \mathbb {G}_1)$, $({\Bbbk }_2, {\mathbb {H}}_2, \mathbb {G}_2)$ *and* $({\Bbbk }_3, {\mathbb {H}}_3, \mathbb {G}_3)$ *be three* $IVBF_{HSS}$*s on* $\mathbb {O}$. *Then*,

(i) $$({\Bbbk }_1, {\mathbb {H}}_1, \mathbb {G}_1) \cup _{e} \big [({\Bbbk }_2, {\mathbb {H}}_2, \mathbb {G}_2) \cup _{e} ({\Bbbk }_3, {\mathbb {H}}_3, \mathbb {G}_3)\big ]$$ = $$\big [({\Bbbk }_1, {\mathbb {H}}_1, \mathbb {G}_1) \cup _{e} ({\Bbbk }_2, {\mathbb {H}}_2, \mathbb {G}_2) \big ] \cup _{e} ({\Bbbk }_3, {\mathbb {H}}_3, \mathbb {G}_3)$$,
(ii) $$({\Bbbk }_1, {\mathbb {H}}_1, \mathbb {G}_1) \cap _{e} \big [({\Bbbk }_2, {\mathbb {H}}_2, \mathbb {G}_2) \cap _{e} ({\Bbbk }_3, {\mathbb {H}}_3, \mathbb {G}_3)\big ]$$ = $$\big [({\Bbbk }_1, {\mathbb {H}}_1, \mathbb {G}_1) \cap _{e} ({\Bbbk }_2, {\mathbb {H}}_2, \mathbb {G}_2)\big ] \cap _{e} ({\Bbbk }_3, {\mathbb {H}}_3, \mathbb {G}_3)$$.

#### *Proof*

(i) The extended union of $({\Bbbk }_1, {\mathbb {H}}_1, \mathbb {G}_1)$ and $(({\Bbbk }_2, {\mathbb {H}}_2, \mathbb {G}_2) \cup _{e} ({\Bbbk }_3, {\mathbb {H}}_3, \mathbb {G}_3)$ is represented as

$$\begin{aligned} ({\Bbbk }_4, {\mathbb {H}}_4, \mathbb {G}_4)=({\Bbbk }_1, {\mathbb {H}}_1, \mathbb {G}_1) \cup _{e} (({\Bbbk }_2, {\mathbb {H}}_2, \mathbb {G}_2) \cup _{e} ({\Bbbk }_3, {\mathbb {H}}_3, \mathbb {G}_3)), \end{aligned}$$

where $\mathbb {G}_4 = \mathbb {G}_1 \cup _{e} ( \mathbb {G}_2 \cup _{e} \mathbb {G}_3)$ and for all ${\mathbb {j}} \in \mathbb {G}_4$,

$$\begin{aligned}{\Bbbk }_4({\mathbb {j}}_{s})= \left\{ \begin{array}{l} {\Bbbk }_1({\mathbb {j}}_{s}), \ \ \ \ \ \ \ \ \ \ \ \ \ \ if \ {\mathbb {j}}_{s} \in \mathbb {G}_1 - ( \mathbb {G}_2 \cup _{e} \mathbb {G}_3) \\ {\Bbbk }_2({\mathbb {j}}_{s}) \cup _{e} {\Bbbk }_3({\mathbb {j}}_{s}), \ \ \ if \ {\mathbb {j}}_{s} \in ( \mathbb {G}_2 \cup _{e} \mathbb {G}_3) - \mathbb {G}_1 \\ {\Bbbk }_1({\mathbb {j}}_{s}) \cup _{e} ({\Bbbk }_2({\mathbb {j}}_{s}) \cup _{e} {\Bbbk }_3({\mathbb {j}}_{s})) \\ if \ {\mathbb {j}}_{s} \in \mathbb {G}_1 \cap _{e} (\mathbb {G}_2 \cup _{e} \mathbb {G}_3 )\\ \end{array}\right\} , \end{aligned}$$

$$\begin{aligned}{\mathbb {H}}_4({\mathbb {j}}_{s})= \left\{ \begin{array}{l} {\mathbb {H}}_1({\mathbb {j}}_{s}), \ \ \ \ \ \ \ \ \ \ \ \ \ \ if \ {\mathbb {j}}_{s} \in \mathbb {G}_1 - ( \mathbb {G}_2 \cup _{e} \mathbb {G}_3) \\ {\mathbb {H}}_2({\mathbb {j}}_{s}) \cup _{e} {\mathbb {H}}_3({\mathbb {j}}_{s}), \ \ \ if \ {\mathbb {j}}_{s} \in ( \mathbb {G}_2 \cup _{e} \mathbb {G}_3) - \mathbb {G}_1 \\ {\mathbb {H}}_1({\mathbb {j}}_{s}) \cap _{e} ({\mathbb {H}}_2({\mathbb {j}}_{s}) \cup _{e} {\mathbb {H}}_3({\mathbb {j}}_{s})) \\ if \ {\mathbb {j}}_{s} \in \mathbb {G}_1 \cap _{e} (\mathbb {G}_2 \cup _{e} \mathbb {G}_3 )\\ \end{array}\right\} , \end{aligned}$$

$$\begin{aligned}{\Bbbk }_4({\mathbb {j}}_{s})= \left\{ \begin{array}{l} {\Bbbk }_1({\mathbb {j}}_{s}), \ \ \ \ \ \ \ \ \ \ \ \ \ \ if \ {\mathbb {j}}_{s} \in \mathbb {G}_1 - ( \mathbb {G}_2 \cup _{e} \mathbb {G}_3) \\ {\Bbbk }_2({\mathbb {j}}_{s}) \cup _{e} {\Bbbk }_3({\mathbb {j}}_{s}), \ \ \ if \ {\mathbb {j}}_{s} \in ( \mathbb {G}_2 \cup _{e} \mathbb {G}_3) - \mathbb {G}_1 \ ({\Bbbk }_1({\mathbb {j}}_{s}) \cup _{e} {\Bbbk }_2({\mathbb {j}}_{s})) \cup _{e} {\Bbbk }_3({\mathbb {j}}_{s}) \\ if \ {\mathbb {j}}_{s} \in \mathbb {G}_1 \cap _{e} (\mathbb {G}_2 \cup _{e} \mathbb {G}_3 )\\ \end{array}\right\} , \end{aligned}$$

and also,

$$\begin{aligned}{\mathbb {H}}_4({\mathbb {j}}_{s})= \left\{ \begin{array}{l} {\mathbb {H}}_1({\mathbb {j}}_{s}), \ \ \ \ \ \ \ \ \ \ \ \ \ \ if \ {\mathbb {j}}_{s} \in \mathbb {G}_1 - ( \mathbb {G}_2 \cup _{e} \mathbb {G}_3) \\ {\mathbb {H}}_2({\mathbb {j}}_{s}) \cup _{e} {\mathbb {H}}_3({\mathbb {j}}_{s}), \ \ \ if \ {\mathbb {j}}_{s} \in ( \mathbb {G}_2 \cup _{e} \mathbb {G}_3) - \mathbb {G}_1 \\ ({\mathbb {H}}_1({\mathbb {j}}_{s}) \cap _{e} {\mathbb {H}}_2({\mathbb {j}}_{s})) \cup _{e} ({\mathbb {H}}_1({\mathbb {j}}_{s}) \cap _{e} {\mathbb {H}}_3({\mathbb {j}}_{s})) \\ if \ {\mathbb {j}}_{s} \in \mathbb {G}_1 \cap _{e} (\mathbb {G}_2 \cup _{e} \mathbb {G}_3 )\\ \end{array}\right\} , \end{aligned}$$

$$\begin{aligned}{\Bbbk }_4({\mathbb {j}}_{s})= \left\{ \begin{array}{l} {\Bbbk }_1({\mathbb {j}}_{s}), \ \ \ \ \ \ \ \ \ \ \ \ \ \ if \ {\mathbb {j}}_{s} \in \mathbb {G}_1 - ( \mathbb {G}_2 \cup _{e} \mathbb {G}_3) \\ {\Bbbk }_2({\mathbb {j}}_{s}) \cup _{e} {\Bbbk }_3({\mathbb {j}}_{s}), \ \ \ if \ {\mathbb {j}}_{s} \in ( \mathbb {G}_2 \cup _{e} \mathbb {G}_3) - \mathbb {G}_1 \\ ({\Bbbk }_1({\mathbb {j}}_{s}) \cup _{e} {\Bbbk }_2({\mathbb {j}}_{s})) \cup _{e} {\Bbbk }_3({\mathbb {j}}_{s}) \\ if \ {\mathbb {j}}_{s} \in \mathbb {G}_1 \cap _{e} (\mathbb {G}_2 \cup _{e} \mathbb {G}_3 )\\ \end{array}\right\} , \end{aligned}$$

$$\begin{aligned} {\mathbb {H}}_4({\mathbb {j}}_{s})= \left\{ \begin{array}{l} {\mathbb {H}}_1({\mathbb {j}}_{s}), \ \ \ \ \ \ \ \ \ \ \ \ \ \ if \ {\mathbb {j}}_{s} \in \mathbb {G}_1 - ( \mathbb {G}_2 \cup _{e} \mathbb {G}_3) \\ {\mathbb {H}}_2({\mathbb {j}}_{s}) \cup _{e} {\mathbb {H}}_3({\mathbb {j}}_{s}), \ \ \ if \ {\mathbb {j}}_{s} \in ( \mathbb {G}_2 \cup _{e} \mathbb {G}_3) - \mathbb {G}_1 \\ {\mathbb {H}}_1({\mathbb {j}}_{s}) \cap _{e} ({\mathbb {H}}_2({\mathbb {j}}_{s}) \cup _{e} ({\mathbb {H}}_1({\mathbb {j}}_{s})) \cap _{e} {\mathbb {H}}_3({\mathbb {j}}_{s}) \\ if \ {\mathbb {j}}_{s} \in \mathbb {G}_1 \cap _{e} (\mathbb {G}_2 \cup _{e} \mathbb {G}_3 )\\ \end{array}\right\} . \end{aligned}$$

Since the commutative property is to be held in $IVBF_{HSS}$, thus:

$$\begin{aligned}{\Bbbk }_4({\mathbb {j}}_{s})= \left\{ \begin{array}{l} {\Bbbk }_1({\mathbb {j}}_{s}), \ \ \ \ \ \ \ \ \ \ \ \ \ \ if \ {\mathbb {j}}_{s} \in \mathbb {G}_1 - ( \mathbb {G}_2 \cup _{e} \mathbb {G}_3) \\ {\Bbbk }_2({\mathbb {j}}_{s}) \cup _{e} {\Bbbk }_3({\mathbb {j}}_{s}), \ \ \ if \ {\mathbb {j}}_{s} \in ( \mathbb {G}_2 \cup _{e} \mathbb {G}_3) - \mathbb {G}_1 \\ ({\Bbbk }_1({\mathbb {j}}_{s}) \cup _{e} {\Bbbk }_2({\mathbb {j}}_{s})) \cup _{e} {\Bbbk }_3({\mathbb {j}}_{s})\\ if \ {\mathbb {j}}_{s} \in \mathbb {G}_1 \cap _{e} (\mathbb {G}_2 \cup _{e} \mathbb {G}_3 )\\ \end{array}\right\} , \end{aligned}$$

$$\begin{aligned}{\mathbb {H}}_4({\mathbb {j}}_{s})= \left\{ \begin{array}{l} {\mathbb {H}}_1({\mathbb {j}}_{s}), \ \ \ \ \ \ \ \ \ \ \ \ \ \ if \ {\mathbb {j}}_{s} \in \mathbb {G}_1 - ( \mathbb {G}_2 \cup _{e} \mathbb {G}_3) \\ {\mathbb {H}}_2({\mathbb {j}}_{s}) \cup _{e} {\mathbb {H}}_3({\mathbb {j}}_{s}), \ \ \ if \ {\mathbb {j}}_{s} \in ( \mathbb {G}_2 \cup _{e} \mathbb {G}_3) - \mathbb {G}_1 \\ {\mathbb {H}}_1({\mathbb {j}}_{s}) \cap _{e} ({\mathbb {H}}_1({\mathbb {j}}_{s}) \cup _{e} ({\mathbb {H}}_2({\mathbb {j}}_{s})) \cap _{e} {\mathbb {H}}_3({\mathbb {j}}_{s}) \\ if \ {\mathbb {j}}_{s} \in \mathbb {G}_1 \cap _{e} (\mathbb {G}_2 \cup _{e} \mathbb {G}_3 )\\ \end{array}\right\} , \end{aligned}$$

$$\begin{aligned}{\Bbbk }_4({\mathbb {j}}_{s})= \left\{ \begin{array}{l} {\Bbbk }_1({\mathbb {j}}_{s}), \ \ \ \ \ \ \ \ \ \ \ \ \ \ if \ {\mathbb {j}}_{s} \in \mathbb {G}_1 - ( \mathbb {G}_2 \cup _{e} \mathbb {G}_3) \\ {\Bbbk }_2({\mathbb {j}}_{s}) \cup _{e} {\Bbbk }_3({\mathbb {j}}_{s}), \ \ \ if \ {\mathbb {j}}_{s} \in ( \mathbb {G}_2 \cup _{e} \mathbb {G}_3) - \mathbb {G}_1 \\ ({\Bbbk }_1({\mathbb {j}}_{s}) \cup _{e} {\Bbbk }_2({\mathbb {j}}_{s})) \cup _{e} {\Bbbk }_3({\mathbb {j}}_{s} \\ if \ {\mathbb {j}}_{s} \in \mathbb {G}_1 \cap _{e} (\mathbb {G}_2 \cup _{e} \mathbb {G}_3 )\\ \end{array}\right\} , \end{aligned}$$

and also,

$$\begin{aligned}{\mathbb {H}}_4({\mathbb {j}}_{s})= \left\{ \begin{array}{l} {\mathbb {H}}_1({\mathbb {j}}_{s}), \ \ \ \ \ \ \ \ \ \ \ \ \ \ if \ {\mathbb {j}}_{s} \in \mathbb {G}_1 - ( \mathbb {G}_2 \cup _{e} \mathbb {G}_3) \\ {\mathbb {H}}_2({\mathbb {j}}_{s}) \cup _{e} {\mathbb {H}}_3({\mathbb {j}}_{s}), \ \ \ if \ {\mathbb {j}}_{s} \in ( \mathbb {G}_2 \cup _{e} \mathbb {G}_3) - \mathbb {G}_1 \\ ({\mathbb {H}}_1({\mathbb {j}}_{s}) \cap _{e} ({\mathbb {H}}_1({\mathbb {j}}_{s})) \cup _{e} ({\mathbb {H}}_2({\mathbb {j}}_{s}) \cap _{e} ({\mathbb {H}}_3({\mathbb {j}}_{s})) \\ if \ {\mathbb {j}}_{s} \in \mathbb {G}_1 \cap _{e} (\mathbb {G}_2 \cup _{e} \mathbb {G}_3 )\\ \end{array}\right\} , \end{aligned}$$

$$\begin{aligned}{\Bbbk }_4({\mathbb {j}}_{s})= \left\{ \begin{array}{l} {\Bbbk }_1({\mathbb {j}}_{s}), \ \ \ \ \ \ \ \ \ \ \ \ \ \ if \ {\mathbb {j}}_{s} \in \mathbb {G}_1 - ( \mathbb {G}_2 \cup _{e} \mathbb {G}_3) \\ {\Bbbk }_2({\mathbb {j}}_{s}) \cup _{e} {\Bbbk }_3({\mathbb {j}}_{s}), \ \ \ if \ {\mathbb {j}}_{s} \in ( \mathbb {G}_2 \cup _{e} \mathbb {G}_3) - \mathbb {G}_1 \\ ({\Bbbk }_1({\mathbb {j}}_{s}) \cup _{e} {\Bbbk }_2({\mathbb {j}}_{s})) \cup _{e} {\Bbbk }_3({\mathbb {j}}_{s})\\ if \ {\mathbb {j}}_{s} \in \mathbb {G}_1 \cap _{e} (\mathbb {G}_2 \cup _{e} \mathbb {G}_3 )\\ \end{array}\right\} , \end{aligned}$$

$$\begin{aligned}{\mathbb {H}}_4({\mathbb {j}}_{s})= \left\{ \begin{array}{l} {\mathbb {H}}_1({\mathbb {j}}_{s}), \ \ \ \ \ \ \ \ \ \ \ \ \ \ if \ {\mathbb {j}}_{s} \in \mathbb {G}_1 - ( \mathbb {G}_2 \cup _{e} \mathbb {G}_3) \\ {\mathbb {H}}_2({\mathbb {j}}_{s}) \cup _{e} {\mathbb {H}}_3({\mathbb {j}}_{s}), \ \ \ if \ {\mathbb {j}}_{s} \in ( \mathbb {G}_2 \cup _{e} \mathbb {G}_3) - \mathbb {G}_1 \\ ({\mathbb {H}}_1({\mathbb {j}}_{s}) \cup _{e} {\mathbb {H}}_2({\mathbb {j}}_{s})) \cap _{e} {\mathbb {H}}_3({\mathbb {j}}_{s}) \\ if \ {\mathbb {j}}_{s} \in \mathbb {G}_1 \cap _{e} (\mathbb {G}_2 \cup _{e} \mathbb {G}_3 )\\ \end{array}\right\} . \end{aligned}$$

This implies

$$\begin{aligned} ({\Bbbk }_4, {\mathbb {H}}_4, \mathbb {G}_) = (({\Bbbk }_1, {\mathbb {H}}_1, \mathbb {G}_1) \cup _{e} ({\Bbbk }_2, {\mathbb {H}}_2, \mathbb {G}_2)) \cup _{e} ({\Bbbk }_3, {\mathbb {H}}_3, \mathbb {G}_3). \end{aligned}$$

Hence, $$({\Bbbk }_1, {\mathbb {H}}_1, \mathbb {G}_1) \cup _{e} (({\Bbbk }_2, {\mathbb {H}}_2, \mathbb {G}_2) \cup _{e} ({\Bbbk }_3, {\mathbb {H}}_3, \mathbb {G}_3))$$ = $$(({\Bbbk }_1, {\mathbb {H}}_1, \mathbb {G}_1) \cup _{e} ({\Bbbk }_2, {\mathbb {H}}_2, \mathbb {G}_2)) \cup _{e} ({\Bbbk }_3, {\mathbb {H}}_3, \mathbb {G}_3)$$.

(ii) The proof is similar to above, and this ends the proof. $\square$

#### **Theorem 4**

*(Distributive property) Let* $({\Bbbk }_1, {\mathbb {H}}_1, \mathbb {G}_1)$, $({\Bbbk }_2, {\mathbb {H}}_2, \mathbb {G}_2)$ *and* $({\Bbbk }_3, {\mathbb {H}}_3, \mathbb {G}_3)$ *be three* $IVBF_{HSS}$*s on* $\mathbb {O}$. *Then*,

(i) $$({\Bbbk }_1, {\mathbb {H}}_1, \mathbb {G}_1) \cap _{e} \big [ ({\Bbbk }_2, {\mathbb {H}}_2, \mathbb {G}_2) \cup _{e} ({\Bbbk }_3, {\mathbb {H}}_3, \mathbb {G}_3)\big ]$$ = $$\big [({\Bbbk }_1, {\mathbb {H}}_1, \mathbb {G}_1) \cap _{e} ({\Bbbk }_2, {\mathbb {H}}_2, \mathbb {G}_2)\big ] \cup _{e} \big [({\Bbbk }_1, {\mathbb {H}}_1, \mathbb {G}_1) \cap _{e} ({\Bbbk }_3, {\mathbb {H}}_3, \mathbb {G}_3)\big ]$$,
(ii) $$({\Bbbk }_1, {\mathbb {H}}_1, \mathbb {G}_1) \cup _{e} \big [({\Bbbk }_2, {\mathbb {H}}_2, \mathbb {G}_2) \cap _{e} ({\Bbbk }_3, {\mathbb {H}}_3, \mathbb {G}_3)\big ]$$ = $$\big [({\Bbbk }_1, {\mathbb {H}}_1, \mathbb {G}_1) \cup _{e} ({\Bbbk }_2, {\mathbb {H}}_2, \mathbb {G}_2)\big ] \cap _{e} \big [({\Bbbk }_1, {\mathbb {H}}_1, \mathbb {G}_1) \cup _{e} ({\Bbbk }_3, {\mathbb {H}}_3, \mathbb {G}_3)\big ]$$.

#### *Proof*

(i) The extended intersection of $({\Bbbk }_1, {\mathbb {H}}_1, \mathbb {G}_1)$ and $(({\Bbbk }_2, {\mathbb {H}}_2, \mathbb {G}_2) \cup _{e} ({\Bbbk }_3, {\mathbb {H}}_3, \mathbb {G}_3)$ is represented by

$$\begin{aligned} ({\Bbbk }_4, {\mathbb {H}}_4, \mathbb {G}_4)=({\Bbbk }_1, {\mathbb {H}}_1, \mathbb {G}_1) \cap _{e} \big [({\Bbbk }_2, {\mathbb {H}}_2, \mathbb {G}_2) \cup _{e} ({\Bbbk }_3, {\mathbb {H}}_3, \mathbb {G}_3)\big ], \end{aligned}$$

where $\mathbb {G}_4 = \mathbb {G}_1 \cup _{e} ( \mathbb {G}_2 \cup _{e} \mathbb {G}_3)$ and for all ${\mathbb {j}} \in \mathbb {G}_4$,

$$\begin{aligned} {\Bbbk }_4({\mathbb {j}}_{s})= \left\{ \begin{array}{l} {\Bbbk }_1({\mathbb {j}}_{s}), \ \ \ \ \ \ \ \ \ \ \ \ \ \ if \ {\mathbb {j}}_{s} \in \mathbb {G}_1 - ( \mathbb {G}_2 \cup _{e} \mathbb {G}_3) \\ {\Bbbk }_2({\mathbb {j}}_{s}) \cup _{e} {\Bbbk }_3({\mathbb {j}}_{s}), \ \ \ if \ {\mathbb {j}}_{s} \in ( \mathbb {G}_2 \cup _{e} \mathbb {G}_3) - \mathbb {G}_1 \\ {\Bbbk }_1({\mathbb {j}}_{s}) \cap _{e} ({\Bbbk }_2({\mathbb {j}}_{s}) \cup _{e} {\Bbbk }_3({\mathbb {j}}_{s})) \\ if \ {\mathbb {j}}_{s} \in \mathbb {G}_1 \cap _{e} (\mathbb {G}_2 \cup _{e} \mathbb {G}_3 )\\ \end{array}\right\} , \end{aligned}$$

$$\begin{aligned} {\mathbb {H}}_4({\mathbb {j}}_{s})= \left\{ \begin{array}{l} {\mathbb {H}}_1({\mathbb {j}}_{s}), \ \ \ \ \ \ \ \ \ \ \ \ \ \ if \ {\mathbb {j}}_{s} \in \mathbb {G}_1 - ( \mathbb {G}_2 \cup _{e} \mathbb {G}_3) \\ {\mathbb {H}}_2({\mathbb {j}}_{s}) \cup _{e} {\mathbb {H}}_3({\mathbb {j}}_{s}), \ \ \ if \ {\mathbb {j}}_{s} \in ( \mathbb {G}_2 \cup _{e} \mathbb {G}_3) - \mathbb {G}_1 \\ {\mathbb {H}}_1({\mathbb {j}}_{s}) \cup _{e} ({\mathbb {H}}_2({\mathbb {j}}_{s}) \cup _{e} {\mathbb {H}}_3({\mathbb {j}}_{s})) \\ if \ {\mathbb {j}}_{s} \in \mathbb {G}_1 \cap _{e} (\mathbb {G}_2 \cup _{e} \mathbb {G}_3 )\\ \end{array}\right\} , \end{aligned}$$

$$\begin{aligned} {\Bbbk }_4({\mathbb {j}}_{s})= \left\{ \begin{array}{l} {\Bbbk }_1({\mathbb {j}}_{s}), \ \ \ \ \ \ \ \ \ \ \ \ \ \ if \ {\mathbb {j}}_{s} \in \mathbb {G}_1 - ( \mathbb {G}_2 \cup _{e} \mathbb {G}_3) \\ {\Bbbk }_2({\mathbb {j}}_{s}) \cup _{e} {\Bbbk }_3({\mathbb {j}}_{s}), \ \ \ if \ {\mathbb {j}}_{s} \in ( \mathbb {G}_2 \cup _{e} \mathbb {G}_3) - \mathbb {G}_1 \\ ({\Bbbk }_1({\mathbb {j}}_{s}) \cap _{e} {\Bbbk }_2({\mathbb {j}}_{s})) \cup _{e} ({\Bbbk }_1({\mathbb {j}}_{s}) \cap _{e} {\Bbbk }_3({\mathbb {j}}_{s})) \\ if \ {\mathbb {j}}_{s} \in \mathbb {G}_1 \cap _{e} (\mathbb {G}_2 \cup _{e} \mathbb {G}_3 )\\ \end{array}\right\} , \end{aligned}$$

$$\begin{aligned} {\mathbb {H}}_4({\mathbb {j}}_{s})= \left\{ \begin{array}{l} {\mathbb {H}}_1({\mathbb {j}}_{s}), \ \ \ \ \ \ \ \ \ \ \ \ \ \ if \ {\mathbb {j}}_{s} \in \mathbb {G}_1 - ( \mathbb {G}_2 \cup _{e} \mathbb {G}_3) \\ {\mathbb {H}}_2({\mathbb {j}}_{s}) \cup _{e} {\mathbb {H}}_3({\mathbb {j}}_{s}), \ \ \ if \ {\mathbb {j}}_{s} \in ( \mathbb {G}_2 \cup _{e} \mathbb {G}_3) - \mathbb {G}_1 \\ ({\mathbb {H}}_1({\mathbb {j}}_{s}) \cap _{e} {\mathbb {H}}_1({\mathbb {j}}_{s})) \cup _{e} ({\mathbb {H}}_2({\mathbb {j}}_{s}) \cup _{e} {\mathbb {H}}_3({\mathbb {j}}_{s})) \\ if \ {\mathbb {j}}_{s} \in \mathbb {G}_1 \cap _{e} (\mathbb {G}_2 \cup _{e} \mathbb {G}_3 )\\ \end{array}\right\} , \end{aligned}$$

$$\begin{aligned}{\Bbbk }_4({\mathbb {j}}_{s})= \left\{ \begin{array}{l} {\Bbbk }_1({\mathbb {j}}_{s}), \ \ \ \ \ \ \ \ \ \ \ \ \ \ if \ {\mathbb {j}}_{s} \in \mathbb {G}_1 - ( \mathbb {G}_2 \cup _{e} \mathbb {G}_3) \\ {\Bbbk }_2({\mathbb {j}}_{s}) \cup _{e} {\Bbbk }_3({\mathbb {j}}_{s}), \ \ \ if \ {\mathbb {j}}_{s} \in ( \mathbb {G}_2 \cup _{e} \mathbb {G}_3) - \mathbb {G}_1 \\ ({\Bbbk }_1({\mathbb {j}}_{s}) \cap _{e} {\Bbbk }_2({\mathbb {j}}_{s})) \cup _{e} ({\Bbbk }_1({\mathbb {j}}_{s}) \cap _{e} {\Bbbk }_3({\mathbb {j}}_{s})) \\ if \ {\mathbb {j}}_{s} \in \mathbb {G}_1 \cap _{e} (\mathbb {G}_2 \cup _{e} \mathbb {G}_3 )\\ \end{array}\right\} , \end{aligned}$$

$$\begin{aligned}{\mathbb {H}}_4({\mathbb {j}}_{s})= \left\{ \begin{array}{l} {\mathbb {H}}_1({\mathbb {j}}_{s}), \ \ \ \ \ \ \ \ \ \ \ \ \ \ if \ {\mathbb {j}}_{s} \in \mathbb {G}_1 - ( \mathbb {G}_2 \cup _{e} \mathbb {G}_3) \\ {\mathbb {H}}_2({\mathbb {j}}_{s}) \cup _{e} {\mathbb {H}}_3({\mathbb {j}}_{s}), \ \ \ if \ {\mathbb {j}}_{s} \in ( \mathbb {G}_2 \cup _{e} \mathbb {G}_3) - \mathbb {G}_1 \\ {\mathbb {H}}_1({\mathbb {j}}_{s}) \cap _{e} ({\mathbb {H}}_1({\mathbb {j}}_{s}) \cup _{e} ({\mathbb {H}}_2({\mathbb {j}}_{s})) \cup _{e} {\mathbb {H}}_3({\mathbb {j}}_{s}) \\ if \ {\mathbb {j}}_{s} \in \mathbb {G}_1 \cap _{e} (\mathbb {G}_2 \cup _{e} \mathbb {G}_3 )\\ \end{array}\right\} , \end{aligned}$$

$$\begin{aligned} {\Bbbk }_4({\mathbb {j}}_{s})= \left\{ \begin{array}{l} {\Bbbk }_1({\mathbb {j}}_{s}), \ \ \ \ \ \ \ \ \ \ \ \ \ \ if \ {\mathbb {j}}_{s} \in \mathbb {G}_1 - ( \mathbb {G}_2 \cup _{e} \mathbb {G}_3) \\ {\Bbbk }_2({\mathbb {j}}_{s}) \cup _{e} {\Bbbk }_3({\mathbb {j}}_{s}), \ \ \ if \ {\mathbb {j}}_{s} \in ( \mathbb {G}_2 \cup _{e} \mathbb {G}_3) - \mathbb {G}_1 \\ ({\Bbbk }_1({\mathbb {j}}_{s}) \cap _{e} {\Bbbk }_2({\mathbb {j}}_{s})) \cup _{e} ({\Bbbk }_1({\mathbb {j}}_{s}) \cap _{e} {\Bbbk }_3({\mathbb {j}}_{s})) \\ if \ {\mathbb {j}}_{s} \in \mathbb {G}_1 \cap _{e} (\mathbb {G}_2 \cup _{e} \mathbb {G}_3 )\\ \end{array}\right\} , \end{aligned}$$

$$\begin{aligned} {\mathbb {H}}_4({\mathbb {j}}_{s})= \left\{ \begin{array}{l} {\mathbb {H}}_1({\mathbb {j}}_{s}), \ \ \ \ \ \ \ \ \ \ \ \ \ \ if \ {\mathbb {j}}_{s} \in \mathbb {G}_1 - ( \mathbb {G}_2 \cup _{e} \mathbb {G}_3) \\ {\mathbb {H}}_2({\mathbb {j}}_{s}) \cup _{e} {\mathbb {H}}_3({\mathbb {j}}_{s}), \ \ \ if \ {\mathbb {j}}_{s} \in ( \mathbb {G}_2 \cup _{e} \mathbb {G}_3) - \mathbb {G}_1 \\ {\mathbb {H}}_1({\mathbb {j}}_{s}) \cap _{e} ({\mathbb {H}}_2({\mathbb {j}}_{s}) \cap _{e} ({\mathbb {H}}_1({\mathbb {j}}_{s})) \cap _{e} {\mathbb {H}}_3({\mathbb {j}}_{s}) \\ if \ {\mathbb {j}}_{s} \in \mathbb {G}_1 \cap _{e} (\mathbb {G}_2 \cup _{e} \mathbb {G}_3 )\\ \end{array}\right\} . \end{aligned}$$

This implies:

$$\begin{aligned} ({\Bbbk }_4, {\mathbb {H}}_4, \mathbb {G}_)=(({\Bbbk }_1, {\mathbb {H}}_1, \mathbb {G}_1) \cap _{e} ({\Bbbk }_2, {\mathbb {H}}_2, \mathbb {G}_2)) \cup _{e} (({\Bbbk }_1, {\mathbb {H}}_1, \mathbb {G}_1) \cap _{e} ({\Bbbk }_3, {\mathbb {H}}_3, \mathbb {G}_3)). \end{aligned}$$

Hence $$({\Bbbk }_1, {\mathbb {H}}_1, \mathbb {G}_1) \cap _{e} (({\Bbbk }_2, {\mathbb {H}}_2, \mathbb {G}_2) \cup _{e} ({\Bbbk }_3, {\mathbb {H}}_3, \mathbb {G}_3))$$ = $$(({\Bbbk }_1, {\mathbb {H}}_1, \mathbb {G}_1) \cap _{e} ({\Bbbk }_2, {\mathbb {H}}_2, \mathbb {G}_2)) \cup _{e} (({\Bbbk }_1, {\mathbb {H}}_1, \mathbb {G}_1) \cap _{e} ({\Bbbk }_3, {\mathbb {H}}_3, \mathbb {G}_3))$$.

(ii) Same as above, and the proof is completed. $\square$

#### **Theorem 5**

*(De Morgan laws) If* $( {\Bbbk }_1, {\mathbb {H}}_1, \mathbb {G}_1)$ *and* $( {\Bbbk }_2, {\mathbb {H}}_2, \mathbb {G}_2)$ *be two* $IVBF_{HSS}$*s on* $\mathbb {O}$, *then*

(i) $$(( {\Bbbk }_1, {\mathbb {H}}_1, \mathbb {G}_1) \cup _{e} ( {\Bbbk }_2, {\mathbb {H}}_2, \mathbb {G}_2))^{'}$$ = $$( {\Bbbk }_1, {\mathbb {H}}_1, \mathbb {G}_1)^{'} \cap _{e} ( {\Bbbk }_2, {\mathbb {H}}_2 \mathbb {G}_2)^{'}$$,
(ii) $$(( {\Bbbk }_1, {\mathbb {H}}_1, \mathbb {G}_1) \cap _{e} ( {\Bbbk }_2, {\mathbb {H}}_2, \mathbb {G}_2))^{'}$$ = $$( {\Bbbk }_1, {\mathbb {H}}_1, \mathbb {G}_1)^{'} \cup _{e} ( {\Bbbk }_2, {\mathbb {H}}_2 \mathbb {G}_2)^{'}$$.

#### *Proof*

(i) We know that $( {\Bbbk }_3, {\mathbb {H}}_3, \mathbb {G}_3)$ = $( {\Bbbk }_1, {\mathbb {H}}_1, \mathbb {G}_1) \cup _{e} ( {\Bbbk }_2, {\mathbb {H}}_2, \mathbb {G}_2)$, where $\mathbb {G}_3 = \mathbb {G}_1 \cup _{e} \mathbb {G}_2$ and

$$\begin{aligned} {\Bbbk }_3({\mathbb {j}}_{s})= \left\{ \begin{array}{l} {\Bbbk }_1({\mathbb {j}}_{s}), \ \ \ \text {if} \ {\mathbb {j}}_{s} \in \mathbb {G}_1 - \mathbb {G}_2 \\ {\Bbbk }_2({\mathbb {j}}_{s}), \ \ \ \text {if} \ {\mathbb {j}}_{s} \in \mathbb {G}_2 - \mathbb {G}_1 \\ {\Bbbk }_1, ({\mathbb {j}}_{s}) \cup _{e} {\Bbbk }_2({\mathbb {j}}_{s}) \ \ \ \text {if} \ {\mathbb {j}}_{s} \in \mathbb {G}_1 \cap _{e} \mathbb {G}_2 \\ \end{array}\right\} , \end{aligned}$$

$$\begin{aligned} {\mathbb {H}}_3({\mathbb {j}}_{s})= \left\{ \begin{array}{l} {\mathbb {H}}_1( {\mathbb {j}}_{s}), \ \ \ \text {if} \ {\mathbb {j}}_{s} \in \mathbb {G}_1 - \mathbb {G}_2 \\ {\mathbb {H}}_2( {\mathbb {j}}_{s}), \ \ \ \text {if} \ {\mathbb {j}}_{s} \in \mathbb {G}_2 - \mathbb {G}_1 \\ {\mathbb {H}}_1( {\mathbb {j}}_{s}) \cap _{e} {\mathbb {H}}_2({\mathbb {j}}_{s}) \ \ \ \text {if} \ {\mathbb {j}}_{s} \in \mathbb {G}_1 \cap _{e} \mathbb {G}_2 \\ \end{array}\right\} , \end{aligned}$$

for all ${\mathbb {j}}_{s} \in \mathbb {G}_3$. Then we have $({\Bbbk }_3, {\mathbb {H}}_3, \mathbb {G}_3)^{'}$ = $(({\Bbbk }_1, {\mathbb {H}}_1, \mathbb {G}_1) \cup _{e} ({\Bbbk }_2, {\mathbb {H}}_2, \mathbb {G}_2))^{'}$ =

$$\begin{aligned} ({\Bbbk }_3({\mathbb {j}}_{s}))^{'}= \left\{ \begin{array}{l} {\mathbb {H}}_1( {\mathbb {j}}_{s}), \ \ \ \text {if} \ {\mathbb {j}}_{s} \in \mathbb {G}_1 - \mathbb {G}_2 \\ {\mathbb {H}}_2( {\mathbb {j}}_{s}), \ \ \ \text {if} \ {\mathbb {j}}_{s} \in \mathbb {G}_2 - \mathbb {G}_1 \\ {\mathbb {H}}_1({\mathbb {j}}_{s}) \cap _{e} {\mathbb {H}}_2({\mathbb {j}}_{s}) \ \ \ \text {if} \ {\mathbb {j}}_{s} \in \mathbb {G}_1 \cap _{e} \mathbb {G}_2 \\ \end{array}\right\} , \end{aligned}$$

$$\begin{aligned} ({\mathbb {H}}_3({\mathbb {j}}_{s}))^{'}= \left\{ \begin{array}{l} {\Bbbk }_1({\mathbb {j}}_{s}) \ \ \ \text {if} \ {\mathbb {j}}_{s} \in \mathbb {G}_1 - \mathbb {G}_2 \\ {\Bbbk }_2({\mathbb {j}}_{s}) \ \ \ \text {if} \ {\mathbb {j}}_{s} \in \mathbb {G}_2 - \mathbb {G}_1 \\ {\Bbbk }_1({\mathbb {j}}_{s}) \cup _{e} {\Bbbk }_2({\mathbb {j}}_{s}) \ \ \ \text {if} \ {\mathbb {j}}_{s} \in \mathbb {G}_1 \cap _{e} \mathbb {G}_2 \\ \end{array}\right\} , \end{aligned}$$

and $$({\Bbbk }_1, {\mathbb {H}}_1, \mathbb {G}_1)^{'} \cap _{e} ({\Bbbk }_2, {\mathbb {H}}_2, \mathbb {G}_2)^{'}$$ =

$$\begin{aligned} ({\Bbbk }_3({\mathbb {j}}_{s}))^{'}= \left\{ \begin{array}{l} {\mathbb {H}}_1( {\mathbb {j}}_{s}), \ \ \ \text {if} \ {\mathbb {j}}_{s} \in \mathbb {G}_1 - \mathbb {G}_2 \\ {\mathbb {H}}_2( {\mathbb {j}}_{s}), \ \ \ \text {if} \ {\mathbb {j}}_{s} \in \mathbb {G}_2 - \mathbb {G}_1 \\ {\mathbb {H}}_1( {\mathbb {j}}_{s}) \cap _{e} {\mathbb {H}}_2( {\mathbb {j}}_{s}) \ \ \ \text {if} \ {\mathbb {j}}_{s} \in \mathbb {G}_1 \cap _{e} \mathbb {G}_2 \\ \end{array}\right\} , \end{aligned}$$

$$\begin{aligned} ({\mathbb {H}}_3({\mathbb {j}}_{s}))^{'}= \left\{ \begin{array}{l} {\Bbbk }_1({\mathbb {j}}_{s}) \ \ \ \text {if} \ {\mathbb {j}}_{s} \in \mathbb {G}_1 - \mathbb {G}_2 \\ {\Bbbk }_2({\mathbb {j}}_{s}) \ \ \ \text {if} \ {\mathbb {j}}_{s} \in \mathbb {G}_2 - \mathbb {G}_1 \\ {\Bbbk }_1({\mathbb {j}}_{s}) \cup _{e} {\Bbbk }_2({\mathbb {j}}_{s}) \ \ \ \text {if} \ {\mathbb {j}}_{s} \in \mathbb {G}_1 \cap _{e} \mathbb {G}_2 \\ \end{array}\right\} . \end{aligned}$$

Hence

$$\begin{aligned} (( {\Bbbk }_1, {\mathbb {H}}_1, \mathbb {G}_1) \cup _{e} ( {\Bbbk }_2, {\mathbb {H}}_2, \mathbb {G}_2))^{'} = ( {\Bbbk }_1, {\mathbb {H}}_1, \mathbb {G}_1)^{'} \cap _{e} ( {\Bbbk }_2, {\mathbb {H}}_2, \mathbb {G}_2)^{'}. \end{aligned}$$

(ii) Since $({\Bbbk }_3, {\mathbb {H}}_3, \mathbb {G}_3)$ = $({\Bbbk }_1, {\mathbb {H}}_1, \mathbb {G}_1) \cap _{e} ({\Bbbk }_2, \mathbb {G}_2)$, where $\mathbb {G}_3 = \mathbb {G}_1 \cup _{e} \mathbb {G}_2$ and

$$\begin{aligned} {\Bbbk }_3({\mathbb {j}}_{s})= \left\{ \begin{array}{l} {\Bbbk }_1({\mathbb {j}}_{s}), \ \ \ \text {if} \ {\mathbb {j}}_{s} \in \mathbb {G}_1 - \mathbb {G}_2 \\ {\Bbbk }_2({\mathbb {j}}_{s}), \ \ \ \text {if} \ {\mathbb {j}}_{s} \in \mathbb {G}_2 - \mathbb {G}_1 \\ {\Bbbk }_1({\mathbb {j}}_{s}) \cap _{e} {\Bbbk }_2({\mathbb {j}}_{s}) \ \ \ \text {if} \ {\mathbb {j}}_{s} \in \mathbb {G}_1 \cap _{e} \mathbb {G}_2 \\ \end{array}\right\} , \end{aligned}$$

$$\begin{aligned} {\mathbb {H}}_3({\mathbb {j}}_{s})= \left\{ \begin{array}{l} {\mathbb {H}}_1( {\mathbb {j}}_{s}), \ \ \ \text {if} \ {\mathbb {j}}_{s} \in \mathbb {G}_1 - \mathbb {G}_2 \\ {\mathbb {H}}_2( {\mathbb {j}}_{s}), \ \ \ \text {if} \ {\mathbb {j}}_{s} \in \mathbb {G}_2 - \mathbb {G}_1 \\ {\mathbb {H}}_1( {\mathbb {j}}_{s}) \cup _{e} {\mathbb {H}}_2({\mathbb {j}}_{s}) \ \ \ \text {if} \ {\mathbb {j}}_{s} \in \mathbb {G}_1 \cap _{e} \mathbb {G}_2 \\ \end{array}\right\} , \end{aligned}$$

then we have $(({\Bbbk }_3, {\mathbb {H}}_3, \mathbb {G}_3))^{'}$ = $(({\Bbbk }_1, {\mathbb {H}}_1, \mathbb {G}_1) \cap _{e} ({\Bbbk }_2, {\mathbb {H}}_2, \mathbb {G}_2))^{'}$ =

$$\begin{aligned} ({\Bbbk }_3({\mathbb {j}}_{s}))^{'}= \left\{ \begin{array}{l} {\mathbb {H}}_1( {\mathbb {j}}_{s}), \ \ \ \text {if} \ {\mathbb {j}}_{s} \in \mathbb {G}_1 - \mathbb {G}_2 \\ {\mathbb {H}}_2( {\mathbb {j}}_{s}), \ \ \ \text {if} \ {\mathbb {j}}_{s} \in \mathbb {G}_2 - \mathbb {G}_1 \\ {\mathbb {H}}_1( {\mathbb {j}}_{s}) \cup _{e} {\mathbb {H}}_2( {\mathbb {j}}_{s}) \ \ \ \text {if} \ {\mathbb {j}}_{s} \in \mathbb {G}_1 \cap _{e} \mathbb {G}_2 \\ \end{array}\right\} , \end{aligned}$$

$$\begin{aligned} ({\mathbb {H}}_3({\mathbb {j}}_{s}))^{'}= \left\{ \begin{array}{l} {\Bbbk }_1({\mathbb {j}}_{s}) \ \ \ \text {if} \ {\mathbb {j}}_{s} \in \mathbb {G}_1 - \mathbb {G}_2 \\ {\Bbbk }_2({\mathbb {j}}_{s}) \ \ \ \text {if} \ {\mathbb {j}}_{s} \in \mathbb {G}_2 - \mathbb {G}_1 \\ {\Bbbk }_1({\mathbb {j}}_{s}) \cap _{e} {\Bbbk }_2({\mathbb {j}}_{s}) \ \ if \ {\mathbb {j}}_{s} \in \mathbb {G}_1 \cap _{e} \mathbb {G}_2 \\ \end{array}\right\} , \end{aligned}$$

and $$({\Bbbk }_1, {\mathbb {H}}_1, \mathbb {G}_1)^{'} \cup _{e} ({\Bbbk }_2, {\mathbb {H}}_2, \mathbb {G}_2)^{'}$$ =

$$\begin{aligned} ({\Bbbk }_3({\mathbb {j}}_{s}))^{'}= \left\{ \begin{array}{l} {\mathbb {H}}_1( {\mathbb {j}}_{s}), \ \ \ \text {if} \ {\mathbb {j}}_{s} \in \mathbb {G}_1 - \mathbb {G}_2 \\ {\mathbb {H}}_2( {\mathbb {j}}_{s}), \ \ \ \text {if} \ {\mathbb {j}}_{s} \in \mathbb {G}_2 - \mathbb {G}_1 \\ {\mathbb {H}}_1( {\mathbb {j}}_{s}) \cup _{e} {\mathbb {H}}_2( {\mathbb {j}}_{s}) \ \ \ \text {if} \ {\mathbb {j}}_{s} \in \mathbb {G}_1 \cap _{e} \mathbb {G}_2 \\ \end{array}\right\} , \end{aligned}$$

$$\begin{aligned} ({\mathbb {H}}_3({\mathbb {j}}_{s}))^{'}= \left\{ \begin{array}{l} {\Bbbk }_1({\mathbb {j}}_{s}) \ \ \ \text {if} \ {\mathbb {j}}_{s} \in \mathbb {G}_1 - \mathbb {G}_2 \\ {\Bbbk }_2({\mathbb {j}}_{s}) \ \ \ \text {if} \ {\mathbb {j}}_{s} \in \mathbb {G}_2 - \mathbb {G}_1 \\ {\Bbbk }_1({\mathbb {j}}_{s}) \cap _{e} {\Bbbk }_2({\mathbb {j}}_{s}) \ \ \ \text {if} \ {\mathbb {j}}_{s} \in \mathbb {G}_1 \cap _{e} \mathbb {G}_2 \\ \end{array}\right\} . \end{aligned}$$

Hence

$$\begin{aligned} (( {\Bbbk }_1, {\mathbb {H}}_1, \mathbb {G}_1) \cap _{e} ( {\Bbbk }_2, {\mathbb {H}}_2, \mathbb {G}_2))^{'} = ({\Bbbk }_1, {\mathbb {H}}_1, \mathbb {G}_1)^{'} \cup _{e} ({\Bbbk }_2, {\mathbb {H}}_2, \mathbb {G}_2)^{'}. \end{aligned}$$

This ends the proof. $\square$

#### **Definition 13**

The OR- operation of $({\Bbbk }_1, {\mathbb {H}}_1, \mathbb {G}_1)$ and $({\Bbbk }_2, {\mathbb {H}}_2, \mathbb {G}_2)$ is defined as

$$\begin{aligned} ({\Bbbk }_5, {\mathbb {H}}_5, \mathbb {G}_5)=({\Bbbk }_1, {\mathbb {H}}_1, \mathbb {G}_1) \vee ({\Bbbk }_2, {\mathbb {H}}_2, \mathbb {G}_2), \end{aligned}$$

where $\mathbb {G}_5 = \mathbb {G}_1 \times \mathbb {G}_2$ and for all $({\mathbb {j}}_s, {\mathbb {j}}_r) \in \mathbb {G}_5$ such that ${\mathbb {j}}_s \in \mathbb {G}_1$ and ${\mathbb {j}}_r \in \mathbb {G}_2$, we have

$$\begin{aligned} {\Bbbk }_{5}({\mathbb {j}}_s, {\mathbb {j}}_r) = {\Bbbk }_{1}({\mathbb {j}}_s) \cup _{e} {\Bbbk }_{2}({\mathbb {j}}_r),~~~~~~{\mathbb {H}}_{5}({\mathbb {j}}_s, {\mathbb {j}}_r) = {\mathbb {H}}_{1}( {\mathbb {j}}_s) \cap _{e} {\mathbb {H}}_{2}( {\mathbb {j}}_r) . \end{aligned}$$

#### *Example 6*

Considering Example 2, let $({\Bbbk }_1, {\mathbb {H}}_1, \mathbb {G}_1)$ and $({\Bbbk }_2, {\mathbb {H}}_2, \mathbb {G}_2)$ be two $IVBF_{HSS}$s. In this case, if

$$\begin{aligned} ({\Bbbk }_5, {\mathbb {H}}_5, \mathbb {G}_5)=({\Bbbk }_1, {\mathbb {H}}_1, \mathbb {G}_1) \vee ({\Bbbk }_2, {\mathbb {H}}_2, \mathbb {G}_2), \end{aligned}$$

then we get

$$({\Bbbk }_5, {\mathbb {H}}_5, \mathbb {G}_5)= \left\{ \begin{array}{l}\ ({\Bbbk }_5, {\mathbb {H}}_5)({\mathbb {j}}_1, {\mathbb {j}}_1)=\{ (\langle {\mathfrak {g}}_{1}, [0.4, 0.9], [-0.7, -0.3] \rangle , \langle {\mathfrak {g}}_{2}, [0.1, 0.6], [-0.4, -0.2]\rangle , \\ \\ \langle {\mathfrak {g}}_{3}, [0.2, 0.7], [-0.6, -0.5]\rangle , \langle {\mathfrak {g}}_{1}, [0.1, 0.5], [-0.7, -0.4]\rangle \},\\ \\ ({\Bbbk }_5, {\mathbb {H}}_5)({\mathbb {j}}_1, {\mathbb {j}}_2)=\{(\langle {\mathfrak {g}}_{1}, [0.2, 0.7], [-0.6, -0.3]\rangle , \langle {\mathfrak {g}}_{2}, [0.1, 0.5], [-0.4, -0.5]\rangle , \\ \\ \langle {\mathfrak {g}}_{3}, [0.1, 0.7], [-0.6, -0.5]\rangle , \langle {\mathfrak {g}}_{1}, [0.2, 1.0], [-0.7, -0.6]\rangle )\}, \\ \\ ({\Bbbk }_5, {\mathbb {H}}_5)({\mathbb {j}}_2, {\mathbb {j}}_1)=\{\langle {\mathfrak {g}}_{1}, [0.3, 0.9], [-0.4, -0.3]\rangle , \langle {\mathfrak {g}}_{2}, [0.1, 0.6], [-0.5, -0.7]\rangle , \\ \\ \langle {\mathfrak {g}}_{3}, [0.2, 0.7], [-0.7, -0.5]\rangle , \langle {\mathfrak {g}}_{4}, [0.1, 0.9], [-0.5, -0.4]\rangle \},\\ \\ ({\Bbbk }_5, {\mathbb {H}}_5)({\mathbb {j}}_2, {\mathbb {j}}_2)=\{\langle {\mathfrak {g}}_{1}, [0.2, 0.6], [-0.4, -0.3]\rangle , \langle {\mathfrak {g}}_{2}, [0.1, 0.5], [-0.9, -0.7]\rangle , \\ \\ \langle {\mathfrak {g}}_{3}, [0.1, 0.7], [-0.8, -0.5]\rangle , \langle {\mathfrak {g}}_{4}, [0.2, 1.0], [-0.5, -0.3] \rangle \}\\ \end{array}\right\} .$$

#### **Definition 14**

The AND-operation of $({\Bbbk }_1, {\mathbb {H}}_1, \mathbb {G}_1)$ and $({\Bbbk }_2, {\mathbb {H}}_2, \mathbb {G}_2)$ is formulated as

$$\begin{aligned} ({\Bbbk }_6, {\mathbb {H}}_6, \mathbb {G}_6)=({\Bbbk }_1, {\mathbb {H}}_1, \mathbb {G}_1) \wedge ({\Bbbk }_2, {\mathbb {H}}_2, \mathbb {G}_2), \end{aligned}$$

where $\mathbb {G}_6 = \mathbb {G}_1 \tilde{\ddot{\times }} \mathbb {G}_2$ and for all $({\mathbb {j}}_s, {\mathbb {j}}_r) \in \mathbb {G}_6$ such that ${\mathbb {j}}_s \in \mathbb {G}_1$ and ${\mathbb {j}}_r \in \mathbb {G}_2$, we have

$$\begin{aligned} {\Bbbk }_{6}({\mathbb {j}}_s, {\mathbb {j}}_r) = {\Bbbk }_{1}({\mathbb {j}}_s) \cap _{e} {\Bbbk }_{2}({\mathbb {j}}_r),~~~~~{\mathbb {H}}_{6}({\mathbb {j}}_s, {\mathbb {j}}_r) = {\mathbb {H}}_{1}( {\mathbb {j}}_s) \cup _{e} {\mathbb {H}}_{2}( {\mathbb {j}}_r). \end{aligned}$$

#### *Example 7*

Considering Example 2, let $({\Bbbk }_1, {\mathbb {H}}_1, \mathbb {G}_1)$ and $({\Bbbk }_2, {\mathbb {H}}_2, \mathbb {G}_2)$ be two $IVBF_{HSS}$s. In this case, if

$$\begin{aligned} ({\Bbbk }_6, {\mathbb {H}}_6, \mathbb {G}_6)=({\Bbbk }_1, {\mathbb {H}}_1, \mathbb {G}_1) \wedge ({\Bbbk }_2, {\mathbb {H}}_2, \mathbb {G}_2), \end{aligned}$$

then we get:

$$({\Bbbk }_6, {\mathbb {H}}_6, \mathbb {G}_6)= \left\{ \begin{array}{l}\ ({\Bbbk }_6, {\mathbb {H}}_6)({\mathbb {j}}_1, {\mathbb {j}}_1)=\{{\mathfrak {g}}_{1}, [0.5,0.7], [-0.8, -0.2]\rangle , \langle {\mathfrak {g}}_{2}, [0.2, 0.3] , [-0.5, -0.1]\rangle , \\ \\ \langle {\mathfrak {g}}_{3}, [0.3, 0.5], [-0.7, -0.4]\rangle , \langle {\mathfrak {g}}_{4}, [0.2, 0.4], [-0.8, -0.3]\rangle \}\\ \\ ({\Bbbk }_6, {\mathbb {H}}_6)({\mathbb {j}}_1, {\mathbb {j}}_2)=\{\langle {\mathfrak {g}}_{1}, [0.5, 0.6], [-0.7, -0.4]\rangle , \langle {\mathfrak {g}}_{2}, [0.2, 0.3], [-1.0, -0.2]\rangle , \\ \\ \langle {\mathfrak {g}}_{3}, [0.3, 0.5], [-0.9, -0.4]\rangle , \langle {\mathfrak {g}}_{4}, [0.2, 0.4], [-0.7, -0.3]\rangle \}, \\ \\ ({\Bbbk }_6, {\mathbb {H}}_6)({\mathbb {j}}_2, {\mathbb {j}}_1)=\{\langle {\mathfrak {g}}_{1}, [0.4, 0.5], [-0.8, -0.2] \rangle , \langle {\mathfrak {g}}_{2}, [0.3, 0.4], [-0.9, -0.1]\rangle , \\ \\ \langle {\mathfrak {g}}_{3}, [0.2, 0.6], [-0.8, -0.4]\rangle , \langle {\mathfrak {g}}_{4}, [0.3, 0.5], [-0.8, -0.3]\rangle \}\\ \\ ({\Bbbk }_6, {\mathbb {H}}_6)({\mathbb {j}}_2, {\mathbb {j}}_2)=\{(\langle {\mathfrak {g}}_{1}, [0.3, 0.5], [-0.6, -0.2]\rangle , \langle {\mathfrak {g}}_{2}, [0.3, 0.4], [-1.0, -0.5]\rangle ,\\ \\ \langle {\mathfrak {g}}_{3}, [0.2, 0.6], [-0.9, -0.4]\rangle , \langle {\mathfrak {g}}_{4}, [0.3, 0.9], [-0.7, -0.6]\}\\ \end{array}\right\} .$$

### Selection of an optimal E-learning solution for personalized learning applications

Recommendation systems play a crucial role in modern digital platforms by providing personalized support to users based on their historical interactions and inferred preferences. These systems are widely used in domains such as e-commerce, e-learning, streaming services, and social networks to enhance user satisfaction and engagement. The core objective of a recommendation system is to filter vast amounts of information and suggest relevant items tailored to each user’s interests. However, traditional recommendation approaches often struggle with issues such as data sparsity, cold start scenarios, and limited adaptability to dynamic user behaviors. To address these limitations, artificial intelligence (AI), particularly machine learning and computational intelligence, has become a natural and effective solution in the development of recommender systems. Techniques such as collaborative filtering, content-based filtering, deep neural networks, clustering, reinforcement learning, and hybrid models have been successfully integrated to improve prediction accuracy and system robustness. These AI-driven approaches not only enhance personalization by learning complex user patterns but also mitigate challenges like a lack of user history and rapidly changing preferences in real-time environments. To further refine the decision-making framework of recommendation systems under uncertain and imprecise information, the concept of $IVBF_{HSS}$ has emerged as a powerful mathematical model. $IVBF_{HSS}$ captures both the positive (liking) and negative (disliking) aspects of user preferences through bipolar information, while interval-valued fuzzy sets account for hesitation and uncertainty. The hypersoft structure supports hierarchical modeling of criteria such as user profiles, item characteristics, and user-item interaction behaviors. This integration enables a more flexible and comprehensive MCDM process, leading to more accurate, interpretable, and context-sensitive recommendation outcomes.

### Statement of the problem

Despite significant advancements in the field of intelligent recommender systems, existing models often face critical challenges when dealing with uncertain, vague, and conflicting user preferences. Traditional methods such as collaborative filtering, content-based filtering, and even hybrid techniques rely heavily on precise user data and predefined preferences, which are not always available or reliable. This creates problems such as the cold-start issue, where new users or items lack sufficient interaction history, and data sparsity, where user-item matrices are too sparse for accurate predictions.

**Fig. 1:**
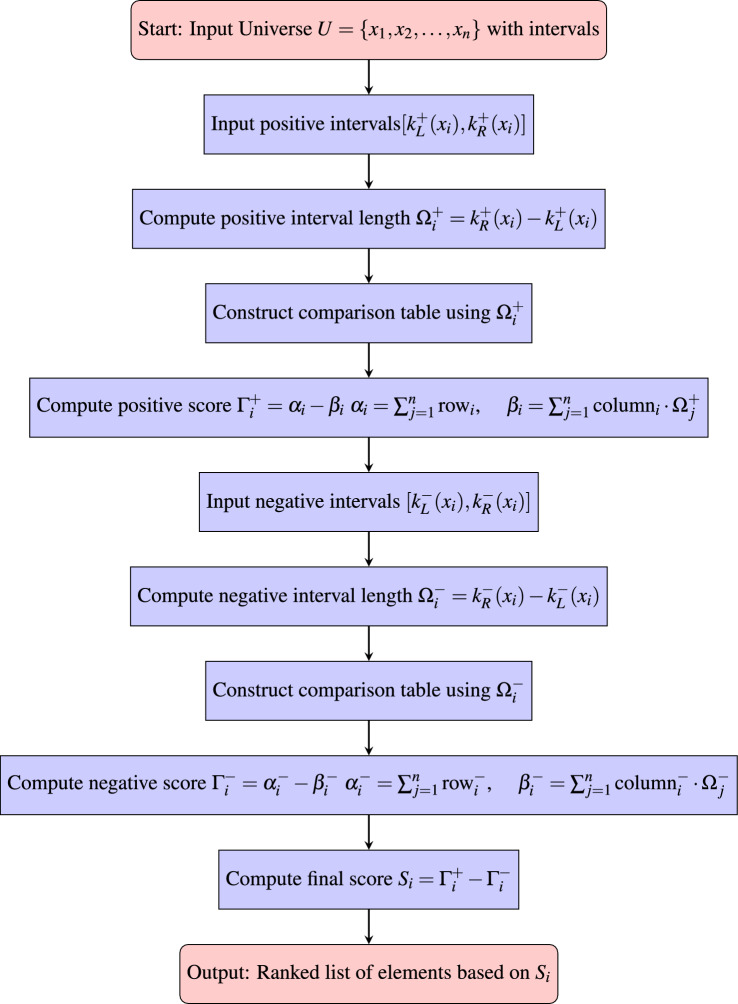
Flowchart of the designed algorithm based on $IVBF_{HSS}$.

Moreover, most conventional models lack the ability to capture both positive and negative user sentiments simultaneously, and they often fail to consider hesitation or neutral stances in decision-making. They are generally limited to flat attribute representations and do not support hierarchical, multi-level criteria structures, which are essential in complex, real-world recommendation scenarios. As a result, such models may generate recommendations that are either inaccurate or irrelevant to the user’s current context. Also, Fig. 1 presents the algorithmic design of the decision support system based on the $IVBF_{HSS}$.

The steps provided in algorithm 1 a systematic framework for analyzing the $IVBF_{HSS}$ model by incorporating positive (likes), negative (dislikes), and neutral/hesitant behaviors through interval-valued fuzzy values. The bipolar nature of the model enables it to capture dual sentiments—such as a scenario where a user likes the price but dislikes the brand. Moreover, the hypersoft structure enhances this model’s capability by supporting multi-level hierarchical decision-making, making it suitable for complex, real-world evaluations. The pseudocode of the following algorithm is provided below:

**Algorithm 1:**
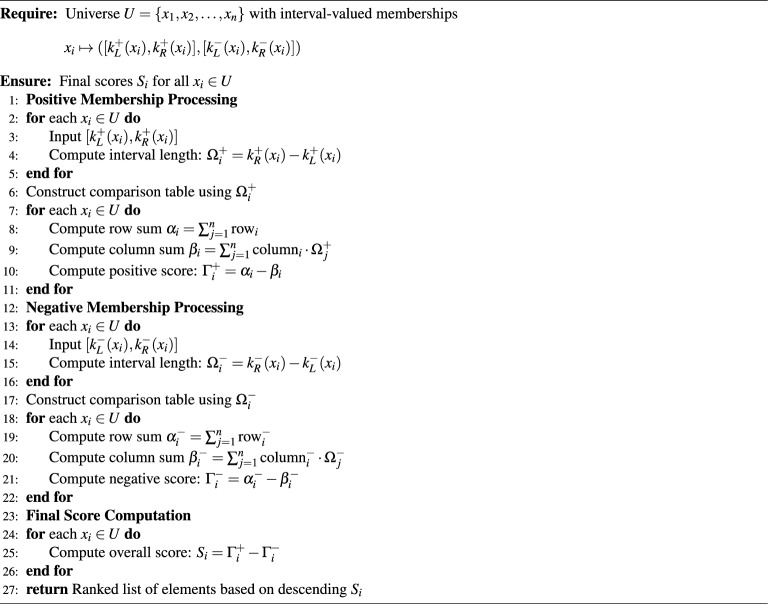
Score computation for $IVBF_{HSS}$.

The following example illustrates how an algorithm using $IVBF_{HSS}$ can facilitate the selection of the best option from a group of in-use e-learning solutions by modeling uncertainty and bipolarity in the data and providing guidance to potential users.

### Numerical example

Suppose that $\mathbb {O}$ = $\{{\mathfrak {g}}_1 =$traditional classroom instruction, ${\mathfrak {g}}_2=$ blended learning, ${\mathfrak {g}}_3=$ self-directed learning, ${\mathfrak {g}}_4=$ informal learning$\}$ be four alternatives to e-learning. Traditional classroom instruction offers face-to-face interaction in a structured environment, while blended learning combines online and in-person instruction for flexibility. Self-directed learning and informal learning empower learners to take control of their education through independent study or practical experiences outside formal settings. $E = \{\textbf{f}_1 = Content, \textbf{f}_2 = Interactivity, \textbf{f}_3 = Feedback, \textbf{f}_4 = Evaluation\}$ is the set of parameters the corresponding attribute values set of every attribute are given by the set $\{\textbf{G}_1, \textbf{G}_2, \textbf{G}_3, \textbf{G}_4\}$, where

$\textbf{G}_1 = \{ b_{11}$ = Relevance, $b_{12}$ = Clarity, $b_{13}$ = Engagement$\}$,

$\textbf{G}_2 = \{ b_{21}$ = Passive, $b_{22}$ = Moderate, $b_{23}$ = Full $\}$,

$\textbf{G}_3 = \{ b_{31}$ = Specificity, $b_{32}$ = Constructiveness, $b_{33}$ = Frequency$\}$,

$\textbf{G}_4 = \{ b_{41}$ = Formative, $b_{42}$ = Summative, $b_{43}$ = Learning outcomes, $b_{44}$ = Learner satisfaction $\}$.

There are one hundred and eight possible cases, but due to some computational limitations and also for the purpose of better explaining the algorithm, we only deal with four cases here, i.e.,

$$\mathbb {G} = \left\{ \begin{array}{l}\ {\mathbb {j}}_1 = ( b_{11}, b_{21},b_{31}, b_{44}),\ \ \ {\mathbb {j}}_2 = (b_{13}, b_{22}, b_{31}, b_{42}),\\ {\mathbb {j}}_3 = (b_{12}, b_{22},b_{31}, b_{41}),\ \ \ {\mathbb {j}}_4 = (b_{11}, b_{23},b_{33}, b_{43}) \\ \end{array}\right\} .$$

The degree of positive membership of $IVBF_{HSS}$ is given by

$$({\Bbbk }_{r}, \mathbb {G})= \left\{ \begin{array}{l} ({\Bbbk }_{r})({\mathbb {j}}_1)=\{\langle {\mathfrak {g}}_{1}, [0.7, 0.8] \rangle , \langle {\mathfrak {g}}_{2}, [0.3, 0.6] \rangle ,\\ \langle {\mathfrak {g}}_{3}, [0.2, 0.5]\rangle , \langle {\mathfrak {g}}_{4}, [0.6, 0.7]\rangle \},\\ {\Bbbk }_{r}({\mathbb {j}}_2)=\{\langle {\mathfrak {g}}_{1}, [0.4, 0.5] \rangle , \langle {\mathfrak {g}}_{2}, [0.1, 0.3]\rangle ,\\ \langle {\mathfrak {g}}_{3}, [0.4, 0.7]\rangle , \langle {\mathfrak {g}}_{4}, [0.3, 0.9]\rangle \}, \\ {\Bbbk }_{r}({\mathbb {j}}_3)=\{ \langle {\mathfrak {g}}_{1}, [0.2, 0.5] \rangle , \langle {\mathfrak {g}}_{2}, [0.2, 0.6]\rangle , \\ \langle {\mathfrak {g}}_{3}, [0.5, 0.7]\rangle , \langle {\mathfrak {g}}_{4}, [0.1, 0.2]\rangle \},\\ {\Bbbk }_{r}({\mathbb {j}}_4)=\{\langle {\mathfrak {g}}_{1}, [0.3, 0.4]\rangle , \langle {\mathfrak {g}}_{2}, [0.3, 0.7]\rangle , \\ \langle {\mathfrak {g}}_{3}, [0.3, 0.5]\rangle , \langle {\mathfrak {g}}_{4}, [0.4, 0.7]\rangle \}\\ \end{array}\right\} .$$

Moreover, the degree of negative membership of $IVBF_{HSS}$ is:

$$({\mathbb {H}}_{r}, \mathbb {G}) = \left\{ \begin{array}{l} {\mathbb {H}}_{r}({\mathbb {j}}_1)=\{\langle {\mathfrak {g}}_{1}, [-0.7,-0.5] \rangle , \langle {\mathfrak {g}}_{2}, [-0.5, -0.4] \rangle , \\ \langle {\mathfrak {g}}_{3}, [-0.7, -0.3]\rangle , \langle {\mathfrak {g}}_{4}, [-0.8, -0.6]\rangle \},\\ {\mathbb {H}}_{r}({\mathbb {j}}_2)=\{\langle {\mathfrak {g}}_{1}, [-0.6, -0.3] \rangle , \langle {\mathfrak {g}}_{2}, [-0.8, -0.2]\rangle , \\ \langle {\mathfrak {g}}_{3}, [-0.9, -0.4]\rangle , \langle {\mathfrak {g}}_{4}, [-0.6, -0.3]\rangle \}, \\ {\mathbb {H}}_{r}({\mathbb {j}}_3)=\{ \langle {\mathfrak {g}}_{1}, [-0.8, -0.4] \rangle , \langle {\mathfrak {g}}_{2}, [-0.6, -0.4]\rangle ,\\ \langle {\mathfrak {g}}_{3}, [-0.9, -0.5]\rangle , \langle {\mathfrak {g}}_{4}, [-0.4, -0.3]\rangle \},\\ {\mathbb {H}}_{r}({\mathbb {j}}_4)=\{\langle {\mathfrak {g}}_{1}, [-0.5, -0.2]\rangle , \langle {\mathfrak {g}}_{2}, [-0.8, -0.5]\rangle , \\ \langle {\mathfrak {g}}_{3}, [-0.3, -0.1]\rangle , \langle {\mathfrak {g}}_{4}, [-0.7, -0.2]\rangle \}\\ \end{array}\right\} .$$

Now, we follow the steps of the aforementioned algorithm:

Step 01. The tabular representation of degree of positive membership of $IVBF_{HSS}$ is shown in Table 2.

**Table 2:** Tabular representation of degree of positive membership of $IVBF_{HSS}$.

$\mathbb {O}$	${\mathbb {j}}_{1}$	${\mathbb {j}}_{2}$	${\mathbb {j}}_{3}$	${\mathbb {j}}_{4}$
${\mathfrak {g}}_1$	[0.7, 0.8]	[0.4, 0.5]	[0.2, 0.5]	[0.3, 0.4]
${\mathfrak {g}}_2$	[0.3, 0.6]	[0.1, 0.3]	[0.2, 0.6]	[0.3, 0.7]
${\mathfrak {g}}_3$	[0.2, 0.5]	[0.4, 0.7]	[0.5, 0.7]	[0.3, 0.5]
${\mathfrak {g}}_4$	[0.6, 0.7]	[0.3, 0.9]	[0.1, 0.2]	[0.4, 0.7]

Step 02. Calculate the length of the fuzzy intervals using the following formula:

$$\begin{aligned} {\Omega _{i} = {\Bbbk }^{{R}^{+}}({\mathfrak {g}}) - {\Bbbk }^{{L}^{+}}({\mathfrak {g}})} \end{aligned}$$

and the results are shown in Table 3.

**Table 3:** Length of the fuzzy intervals.

$\mathbb {O}$	${\Omega _1}^{+}$	$\Omega _{2}^{+}$	$\Omega _{3}^{+}$	$\Omega _{4}^{+}$
${\mathfrak {g}}_1$	0.1	0.1	0.3	0.1
${\mathfrak {g}}_2$	0.3	0.2	0.4	0.4
${\mathfrak {g}}_3$	0.3	0.3	0.2	0.2
${\mathfrak {g}}_4$	0.1	0.6	0.1	0.3

Step 03. The Row sum $\alpha = \{ \text {sum of the horizontal rows}~\Omega _{i}\}$, and the Column sum $\beta = \{ \text {sum of the vertical columns}~ \Omega _{j}\}$ and the Score function of degree of positive membership of $IVBF_{HSS}$ can be calculated using the formula $\Gamma = \alpha - \beta$, and the results are shown in Table 4.

**Table 4:** Score function of degree of positive membership of $IVBF_{HSS}$.

$\mathbb {O}$	$\alpha ^{+}$	$\beta ^{+}$	$\Gamma ^{+}$
${\mathfrak {g}}_1$	0.6	0.8	-0.2
${\mathfrak {g}}_2$	1.3	1.2	0.1
${\mathfrak {g}}_3$	1.0	1.0	0.0
${\mathfrak {g}}_4$	1.1	1.0	0.1

Step 04. The tabular representation of degree of negative membership of $IVBF_{HSS}$ is shown in Table 5.

**Table 5:** Tabular representation of degree of negative membership of $IVBF_{HSS}$.

$\mathbb {O}$	${\mathbb {j}}_{1}$	${\mathbb {j}}_{2}$	${\mathbb {j}}_{3}$	${\mathbb {j}}_{4}$
${\mathfrak {g}}_1$	[– 0.7, – 0.5]	[– 0.6, – 0.3]	[– 0.8, – 0.4]	[– 0.5, – 0.2]
${\mathfrak {g}}_2$	[– 0.5, – 0.4]	[– 0.8, – 0.2]	[– 0.6, – 0.4]	[– 0.8, – 0.5]
${\mathfrak {g}}_3$	[– 0.7, – 0.3]	[– 0.9, – 0.4]	[– 0.9, – 0.5]	[– 0.3, – 0.1]
${\mathfrak {g}}_4$	[– 0.8, – 0.6]	[– 0.6, – 0.3]	[– 0.4, – 0.3]	[– 0.7, – 0.2]

Step 05. The length of the fuzzy intervals obtained by using formula ${\Omega _{i} = {\Bbbk }^{{R}^{-}}({\mathfrak {g}}) - {\Bbbk }^{{L}^{-}}({\mathfrak {g}})}$ is shown in Table 6.

**Table 6:** The length of the fuzzy intervals.

$\mathbb {O}$	${\Omega _1}^{-}$	${\Omega _2}^{-}$	${\Omega _3}^{-}$	${\Omega _4}^{-}$
${\mathfrak {g}}_1$	0.2	0.3	0.4	0.3
${\mathfrak {g}}_2$	0.1	0.6	0.2	0.3
${\mathfrak {g}}_3$	0.4	0.5	0.4	0.2
${\mathfrak {g}}_4$	0.2	0.3	0.1	0.5

Step 06. Values of $\overline{\Gamma }$ is shown in Table 7.

**Table 7:** Score function of degree of negative membership of $IVBF_{HSS}$.

$\mathbb {O}$	${\alpha }^{-}$	${\beta }^{-}$	${\Gamma }^{-}$
${\mathfrak {g}}_1$	1.2	0.7	0.5
${\mathfrak {g}}_2$	1.2	1.7	– 0.5
${\mathfrak {g}}_3$	1.5	1.1	0.4
${\mathfrak {g}}_4$	1.1	1.3	– 0.2

Step 07. Final results, which are useful for ranking, are shown in Table 8.

**Table 8:** Final score.

$\mathbb {O}$	$\Gamma ^{+}$	${\Gamma }^{-}$	*S*
${\mathfrak {g}}_1$	– 0.2	0.5	– 0.7
${\mathfrak {g}}_2$	0.1	– 0.5	0.6
${\mathfrak {g}}_3$	0.0	0.4	– 0.4
${\mathfrak {g}}_4$	0.1	– 0.2	– 0.1

From Table 8, we notice that:

$$\begin{aligned} {\mathfrak {g}}_2 \ \ \widetilde{\succeq }\ \ {\mathfrak {g}}_4 \ \ \widetilde{\succeq }\ \ {\mathfrak {g}}_3 \ \ \widetilde{\succeq }\ \ {\mathfrak {g}}_1. \end{aligned}$$

The software with the maximum score is chosen from Table 8, which is ${\mathfrak {g}}_2$. So, ${\mathfrak {g}}_2$ is the best choice because it achieved the maximum score and is considered the best alternative during the decision-making process. The process illustrated above uses the concepts introduced earlier in the paper. By incorporating bipolarity into the fuzzy decision-making process, it is possible to analyze the complex relationships and opposing forces involved in a decision and make a more informed and effective decision, as illustrated by the example above.

### Comparative analysis of the designed structure

To validate the effectiveness of the proposed recommendation system based on $IVBF_{HSS}$, we performed a set of comparative experiments shown in Tables 9 and 10. Precision, recall, and F1 score values were calculated by first calculating the lengths of the membership functions valued by intervals for each element $g_i$ across all subattributes $\gamma _j$. The true positive value (TP) was taken as the sum of these interval lengths. To test sensitivity, we introduced a uniform perturbation of ±0.1: 1. In the +0.1 case, the predicted membership intervals are slightly overestimated, creating false positives (FP) equal to 0.1 of the TP, while FN = 0. 2. In the -0.1 case, the intervals are underestimated, creating false negatives (FN) equal to 0.1 of TP, while FP = 0. Finally, precision ($\frac{TP}{TP+FP}$), recall ($\frac{TP}{TP+FN}$), and the F1-score (harmonic mean of precision and recall) were calculated. Because of the symmetric ±0.1 perturbation, all variants (fuzzy set, soft set, FBHSS, and $IVBF_{HSS}$) produced precision = 0.91, recall = 0.91–1.00, and F1 = 0.95. These experiments aim to evaluate the system against well-established baseline algorithms and demonstrate the superiority of $IVBF_{HSS}$ in handling uncertainty, bipolarity, and interval-valued data in e-learning recommendations. We used a data set consisting of user preferences, learning material attributes, and ratings, represented in the hypersoft bipolar fuzzy format with interval values. $IVBF_{HSS}$ produces rankings that better match actual student preferences due to the incorporation of bipolar information and interval-valued information. The $IVBF_{HSS}$-based recommendation system outperforms traditional CF and Fuzzy TOPSIS due to:

1. Handling interval-valued memberships, which model uncertainty.
2. Incorporating bipolar information, representing both positive and negative preferences.
3. Aggregating sub-attribute information via hypersoft operations, which improves decision accuracy in multi-criteria e-learning contexts.

Experimental evidence strongly supports the superiority of IVBFHSS for personalized e-learning recommendation.

**Table 9:** Sensitivity analysis.

Set type	Perturbation	TP	FP	FN	Precision	Recall	F1-score
Fuzzy Set	+ 0.1	7.3	0.73	0	0.909	1.0	0.952
Fuzzy Set	– 0.1	7.3	0	0.73	1.0	0.909	0.952
Soft Set	+ 0.1	4.0	0.4	0	0.909	1.0	0.952
Soft Set	– 0.1	4.0	0	0.4	1.0	0.909	0.952
FBHSS Positive	+ 0.1	4.0	0.4	0	0.909	1.0	0.952
FBHSS Positive	– 0.1	4.0	0	0.4	1.0	0.909	0.952
FBHSS Negative	+ 0.1	5.0	0.5	0	0.909	1.0	0.952
FBHSS Negative	– 0.1	5.0	0	0.5	1.0	0.909	0.952
$IVBF_{HSS}$	+ 0.1	9.0	0.9	0	0.909	1.0	0.952
$IVBF_{HSS}$	– 0.1	9.0	0	0.9	1.0	0.909	0.952

**Table 10:** Comparison with baseline algorithm.

Algorithm	Precision	Recall	F1-score	Coverage
Collaborative filtering	0.78	0.72	0.75	0.65
Fuzzy TOPSIS	0.84	0.79	0.81	0.70
$IVBF_{HSS}$	0.91	0.88	0.89	0.82

The comparative analysis in Table 11 reveals that while previous mathematical structures each address certain complexities in decision making, they fail to capture the full spectrum of hypersoft, bipolar, and interval valued fuzzy environments simultaneously. The proposed $IVBF_{HSS}$ effectively integrates these features, thereby eliminating the identified limitations and providing a more robust and comprehensive framework for complex decision-making problems.

**Table 11:** Comparative analysis of soft, fuzzy, bipolar, and hypersoft set variants motivating $IVBF_{HSS}$.

Mathematical structure	Benefits	Limitations
SS^3^	Consideration of numerous parameterized families of sets with multiple decision attributes	Single set of parameters
HSS^32^	Division of parameter set into disjoint sets with sub-parametric values	Inability to distinguish between bipolar sets of decision attributes
BSS^24^	Handling of symmetrically opposed attribute sets	Inability to handle multi-argument function
FBSS^25^	Management of bipolar soft information in a fuzzy environment	Neglect of Cartesian product of sub- parametric valued disjoint classes
BHSS^48^	Dealing with multi-argument function with bipolar information	Inability to handle information with IVBF membership degree
The proposed hybrid method $IVBF_{HSS}$	Focus on hypersoft bipolar information in IVBF environment	Overcoming all the limitations

It is important to recognize that the present evaluation of the IV-BFHSS framework was conducted exclusively on synthetic datasets. While synthetic data enables controlled benchmarking and technical validation, it does not fully replicate the diversity, complexity, and unpredictability of real-world e-learning platforms. As a result, the experimental outcomes may overestimate robustness and generalizability. Additionally, the current approach utilizes expert-defined interval inputs to represent learner preferences, which, though effective for modeling uncertainty, may introduce subjectivity and affect reproducibility and fairness in large-scale or cross-domain applications. These limitations underscore the need for future work involving empirical validation on authentic e-learning datasets and the development of automated, data-driven interval estimation techniques to minimize expert bias and enhance reliability.

## Conclusions

This paper introduces a highly versatile Interval-Valued Bipolar Fuzzy Hypersoft Set structure as a combination of both Bipolar Fuzzy Hypersoft Set and Interval-Valued Bipolar Fuzzy Set structures. The proposed approach addresses key limitations of traditional models by simultaneously incorporating uncertainty, hesitation, and bipolar preferences, while also enabling the decomposition of attributes into multiple sub-attributes for more fine-grained analysis. The bipolar preferences allow for the incorporation of positive and negative learner feedback, making it more suitable for decision-making applications. The essential properties and basic set-theoretic operations of $IVBF_{HSS}$ are thoroughly discussed in the work. This includes defining membership and non-membership degrees within intervals to capture the uncertainty inherent in decision attributes. We introduce new set notions and theoretical operations tailored to $IVBF_{HSS}$, including operations such as restricted union, extended union, intersection, AND, OR operation, etc. Additionally, in the paper, the commutative, associative, distributive, and De-morgan laws are examined, which ensure a comprehensive analysis. In this article, the objective of selecting the best alternative in e-learning, such as identifying the most suitable instructional method, can effectively be formulated as an MADM problem. This approach allows for the systematic evaluation of various alternatives based on multiple parameters and sub-parameters, enabling a rational and well-informed decision. In this context, a decisive supporting mechanism is established through the use of a robust and reliable algorithm that can effectively represent the uncertainty and bipolarity present in the data, allowing decision-makers to make informed choices while considering multiple attributes simultaneously. The presented approach offers a single, comprehensive structure that exhibits greater flexibility compared to existing approaches. However, it’s important to acknowledge certain limitations and implications associated with this framework. Notably, the framework currently lacks support for intuitionistic fuzzy and neutrosophic settings. As a result, future modifications may be necessary to adapt the framework for use in these contexts. Additionally, the framework does require interval-valued inputs for both positive and negative membership degrees. While suitable for capturing uncertainty, the accurate specification of these intervals depends heavily on expert judgment or predefined mapping schemes, which may introduce subjectivity. The $IVBF_{HSS}$ framework lays a strong foundation for uncertain, bipolar, sub-attribute-aware decision-making in complex domains like e-learning. Future research will focus on validating the system using large-scale, real-world e-learning data and developing automated, machine learning-based methods for interval estimation to foster more robust and fair practical deployment. The proposed future work transforms it from a theoretical construct into a practical, adaptive, and scalable decision support system, fully addressing current limitations and paving the way for next-generation personalized education technologies.

## Supplementary Information

Supplementary Information.

## Data Availability

All data generated or analysed during this study is included in the article.

## Code availability

All code used to produce the computational results is provided in the Supplementary Information in the file titled “IVBFHSS.xlxs”.
